# Providing more balanced information on the harms and benefits of cervical cancer screening: A randomized survey among US and Norwegian women

**DOI:** 10.1016/j.pmedr.2021.101452

**Published:** 2021-06-23

**Authors:** P.R. Cyr, K. Pedersen, A.L. Iyer, M.K. Bundorf, J.D. Goldhaber-Fiebert, D. Gyrd-Hansen, I.S. Kristiansen, E.A. Burger

**Affiliations:** a Department of Global Health and Community Medicine, Institute of Health and Society, University of Oslo, P.O. Box 1039 Blindern, 0318 Oslo, Norway; b Department of Health Management and Health Economics, Institute of Health and Society, University of Oslo, P.O. Box 1039 Blindern, 0318 Oslo, Norway; c Stanford School of Public Policy, Duke University, Durham, NC 27708 and NBER, United States; d Danish Centre for Health Economic, Department of Public Health, University of Southern Denmark, J.B. Winsløws Vej 9B, 1^st^ Floor, 5000, Odense C, Denmark; e Centers for Health Policy and Primary Care and Outcomes Research, Stanford Health Policy, Stanford University, Stanford, CA, United States

**Keywords:** Cervical cancer, Cancer screening, Patient education, Screening participation, Informed decision-making, Risk information

## Abstract

We aimed to identify how additional information about benefits and harms of cervical cancer (CC) screening impacted intention to participate in screening, what type of information on harms women preferred receiving, from whom, and whether it differed between two national healthcare settings. We conducted a survey that randomized screen-eligible women in the United States (n = 1084) and Norway (n = 1060) into four groups according to the timing of introducing additional information. We found that additional information did not significantly impact stated intentions-to-participate in screening or follow-up testing in either country; however, the proportion of Norwegian women stating uncertainty about seeking precancer treatment increased from 7.9% to 14.3% (p = 0.012). Women reported strong system-specific preferences for sources of information: Norwegians (59%) preferred it come from a national public health agency while Americans (59%) preferred it come from a specialist care provider. Regression models revealed having a prior Pap-test was the most important predictor of intentions-to-participate in both countries, while having lower income reduced the probabilities of intentions-to-follow-up and seek precancer treatment among U.S. women. These results suggest that additional information on harms is unlikely to reduce participation in CC screening but could increase decision uncertainty to seek treatment. Providing unbiased information would improve on the ethical principle of respect for autonomy and self-determination. However, the clinical impact of additional information on women’s understanding of the trade-offs involved with CC screening should be investigated. Future studies should also consider country-specific socioeconomic barriers to screening if communication re-design initiatives aim to improve CC screening participation.

## Highlights

• Additional information did not impact intentions to participate in CC screening.
• Additional information increased uncertainty to seek precancer treatment in Norway.
• Women reported strong system-specific preferences for sources of information.
• Having a prior Pap-test was an important predictor of intentions-to-participate.
• Socioeconomic factors influenced follow-up intentions in the U.S. but not in Norway.

## 1 Background

Cervical cancer (CC) screening has contributed to significant reductions in the burden of CC in countries such as the United States (U.S.) and Norway. Despite the substantial benefits of screening, i.e., reductions in incidence and mortality (Bray et al., 2005, Peirson et al., 2013), screening also involves potential harms to women. For example, most women who undergo treatment to remove a high-grade precancer would never have developed invasive CC had it been left untreated; however, at the time of treatment, it is impossible to distinguish between women who would develop cancer from those who would not (McCredie et al., 2008, Ostor, 1993). Side-effects from follow-up procedures can include infection or bleeding from punch biopsies or conization, and adverse pregnancy-related outcomes (Jin et al., 2014, Kyrgiou et al., 2016, Sharp et al., 2009). In addition, CC screening may induce screen-related anxiety (Stout et al., 2008). Therefore, screening inherently involves trade-offs in health benefits and potential harms. Importantly, a study by Kolthoff et al. (2016) found that information provided to women in 10 developed countries were biased towards inciting participation and often neglected to include information on harms such as overdiagnosis and overtreatment.

The organization of CC screening in the U.S. and Norway is vastly different. In the U.S., national screening recommendations are set by several different organizations (Saslow et al., 2012) and it is at the discretion of individual providers to follow and track patient adherence. In contrast, in Norway, the CC Screening Program is managed centrally by the Cancer Registry. Norwegian women who have not participated in routine screening or did not follow-up after an abnormal result are identified in the database and receive reminder letters. Screening coverage within a 5-year period is approximately 80% in Norway (Cancer Registry of Norway, 2020). Similar coverage is reported by the New Mexico HPV Pap Registry (the only existing lab-based population-based CC screening registry in the U.S.) (Cuzick et al., 2014). Although U.S coverage varies by setting, the New Mexico CC incidence and mortality largely mimics that of the broader U.S. population (Kim et al., 2015).

National policy objectives and guidelines increasingly incorporate patient involvement, informed-choice and shared decision-making (Norwegian Ministry of Health and Care Services, 2015, Centers for Medicare and Medicaid Services, 2015, Moyer, 2014). Greater emphasis is being placed on considering patient preferences; failing to provide unbiased information reduces a woman’s ability to make informed choices about healthcare (Hersch et al., 2015, Kolthoff et al., 2016). As countries increase focus on prevention, it is important to consider how to engage with individuals who are invited to prevention programs. A recent study found that Dutch women preferred to receive their breast cancer risk estimate and tailored recommendations for screening (Rainey et al., 2020).

Little evidence exists to guide policymakers on what type of information women would prefer on CC screening, and through what channels they would like to receive it. In addition, little is known on how women’s screening decisions are affected by the information provided to them and whether preferences differ across diverse healthcare systems. Understanding how information on harms and benefits influence participation and whether the timing and preferred source of information changes participation outcomes would be useful for policymakers when designing letters and other written communications inviting women to participate in screening.

Therefore, we first aimed to identify how information presented at different stages of the screening process about benefits and harms impacts women’s intention to participate in the U.S. and Norway. We then explored women’s preferences as to the type of information they receive and from whom they prefer to receive it. Socioeconomic and other factors have been shown to impact women’s participation in screening (Johnson et al., 2008, Labeit et al., 2013). Therefore, as a secondary aim, we explored whether women’s stated intentions to participate in screening in our survey reflected these previously reported patterns.

## 2 Methods

### 2.1 Study design

In 2013, we conducted a randomized, web-based survey in Norway and the U.S. utilizing TNS Gallup’s active internet panel of >50,000 Norwegians and Gfk’s KnowledgePanel® of 55,000 U.S. individuals. The study materials and design were approved by the Regional Committee for Medical and Health Research Ethics, South East, Norway (2012/2158/REK Southeast B) and the institutional review board at Stanford University (IRB: 6208).

### 2.2 Study population

Both surveys targeted a representative sample of screen-eligible women based on national age-specific screening guidelines (women aged 25–69 years in Norway and aged 21–65 years in the U.S.; Appendix, Tables A1/A2). We sent the survey invitation with a short description of the study’s objective to 2626 women in Norway and 2594 women in the U.S. 1275 (49%) and 1331 (51%) of women opened the survey invitation in Norway and the U.S., respectively (Appendix, Fig. A1). Recruitment ceased by deactivating the website when a pre-determined number of maximum participants had responded. Informed consent was obtained from all participants.

### 2.3 Survey design

**Fig. 1:**
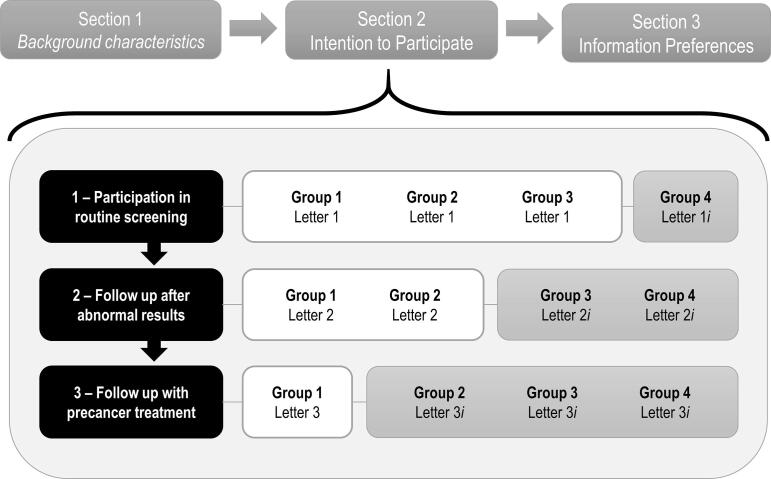
Schematic of the survey structure. Following consent, the survey was divided in 3 sections with a total of 28 questions. For the primary analysis, women in each country were collapsed at each stage of the simplified screening into two main groups: women that had received additional information about screening harms and benefits (lower panel, grey bars), and woman who had not (lower panel, white bars). Group 1 constituted the control arm and received no additional information (letters 1–2-3). Group 2 received expanded information at the point of a recommendation to seek treatment (letters 1–2-3*i*), Group 3 at the point of a recommendation to follow-up abnormal results (letters 1-2*i*-3*i*), and Group 4 at the initial routine screening invitation (letters 1*i*-2*i*-3*i*).

The survey design for the Norwegian survey has been described previously and was structured identically in the U.S. (Iyer et al., 2019). The survey was divided in three sections: (1) background and baseline knowledge, (2) screening participation, and precancer treatment intentions and (3) information preferences, with a total of 28 questions (Fig. 1).

Section 2 of the survey presented CC screening as a simplified three-step process. Each step was accompanied by an information letter. The first letter (‘1′) included an initial invitation to routine CC screening and recommendation to have a Pap test; the second letter (‘2′) included a reminder to follow-up after a hypothetical abnormal test result; and the third letter (‘3′) included a recommendation to seek surgical treatment following hypothetical detection of a high-grade precancer. To limit the burden of information on participants, the simplified steps excluded details regarding HPV and cytology co-testing and follow-ups with a colposcopy/biopsy that usually precede treatment. For the Norwegian survey, letters 1 and 2 were based on the text of the Norwegian CC Screening Program 2012 letter, albeit simplifications due to constraints of the survey format. Recommendations for treatment are generally sent by healthcare providers, not the program. Therefore, we created letter 3 based on similar wording used elsewhere by the screening program. For the U.S., given that screening is not organized by a single agency, we modified the Norwegian letters to reflect the 2013 CDC-recommended guidelines for routine CC screening.

#### 2.3.1 Randomization

To assess the potential impact of providing additional information on the harms related to CC screening, we introduced randomization in section 2 of the survey. We developed a second version of each letter (i.e., ‘*i*’ version). Version ‘*i*’ of each letter provided additional information.

To avoid survey fatigue linked to excessive information, we limited the additional information to 4 topics presented in a way that facilitates understanding among the target audience: (1) the possibility of experiencing anxiety from screening procedures, (2) the potential need for additional testing, (3) the likelihood of precancer lesion regression without treatment, and (4) the potential link between surgical treatment and adverse pregnancy outcomes (Appendix). We randomized women into one of four groups, which varied the point in the screening process at which they received the letters with expanded information (Fig. 1). Each question in section 2 was posed independently of prior responses.

### 2.4 Study outcomes

The primary study endpoints were the stated intentions to follow recommendations at each step of the screening process and response certainty. Secondary outcomes were preferences as to the timing and source of information.

### 2.5 Sample size and statistical analyses

The recruitment goal was based on pilot work conducted in a previous and related study (Burger et al., 2014) in which 92% of respondents ‘intended to participate’ in CC screening with a mean response score of 8.31 (10-point Likert scale of 1 = ‘I will absolutely not participate’ and 10 = ‘I will absolutely participate’). We hypothesized that a decrease of 0.5 when information on screening-related harms was provided would be clinically meaningful. Assuming 80% power at a significance level of 5%, we calculated that we would need at least 175 respondents in each of the four groups. The sample size was increased based on available funding and to allow for potential incomplete responses.

Although hysterectomy was not an exclusion criterion to complete the survey, we excluded these women from analyses of survey section 2 (intention to participate in screening), as they are not recommended screening in either country.

We undertook a three-part analysis to evaluate: 1) whether providing additional information about the potential harms of CC screening influenced intention to participate and follow recommendations, 2) women’s preferences for the information they receive and 3) whether socioeconomic and other factors influenced women’s intention to participate in CC screening.

For the analyses, the four randomized groups were collapsed into two groups according to whether the group had received additional information about harms or not. We calculated proportions for each question with categorical endpoints including ‘intend to participate’, ‘do not intend to participate’ and ‘don’t know’. We used chi-squared tests to assess statistical differences in stated intentions to participate or attend follow-up/treatment recommendations. Head-to-head comparison of the 3 response categories were also done using a Bonferroni correction. Women’s response certainty was calculated as the average on a 1–10 Likert scale and a non-parametric test (Kruskal-Wallis) was used to test for differences between groups. We used the chi-squared test to assess differences between women’s preferred source of information.

We then explored how socio-economic and other factors may influence stated intentions to participate. Given the different cultural context and structure of healthcare systems in the U.S. and Norway, we explored outcomes for each country independently. We recoded the dependent variable as a dichotomous outcome (0 = will not participate or don’t know, 1 = will participate) and used binomial logistic regressions to ease interpretation. The variables included were chosen based on (1) a priori decision based on their relevance to the dependent variable and (2) their statistical significance (each variable was statistically significant in at least one country, for at least one participation question, in at least one univariate model). We present the effect of the independent variables in an adjusted multivariable model that accounts for the additional information women received and relevant covariates known to be predictors of the outcome (i.e. household income, age, marital status, level of education, knowledge of HPV as a main cause of CC, an indicator for culture (ethnicity in the U.S. or born in Norway), plans of a future pregnancy, a prior Pap test (Challier et al., 2000, Johnson et al., 2008, Moser et al., 2009, Robb et al., 2010, Waller et al., 2012, Labeit et al., 2013, Bernard et al., 2013)). The unadjusted effect of each variable in univariate models is found in Appendix (Table A6). The average marginal effects, expressed as a percentage point (PP), was calculated to enable reading variables on their original scale and can be interpreted as the average change in probability when compared to the probability at baseline.

All data were de-identified prior to the analyses. To reduce the effect of non-responses and non-coverage bias in the web panels, we used the GfK- and TNS Gallup-constructed post-stratification weights (i.e., weighted distribution of responses). These weights were computed from population demographic and geographic distributional data. The benchmark distributions used in the adjustment in Norway were age, living location and education, whereas in the U.S., age, ethnic background, education, household income, census region, metropolitan area (yes/no) and internet access (yes/no) were used (Appendix).

All data were analyzed using SPSS, version 20 and STATA, release 12.

## 3 Results

### 3.1 Study population

A total of 1060 Norwegian and 1084 U.S. women responded to the survey. The mean age was 45.8 years in Norway and 43.1 years in the U.S. The baseline demographic characteristics were evenly distributed among the randomized groups after the weight application (Appendix, Tables A1/A2).

### 3.2 Baseline knowledge

Among respondents, 88.9% of Norwegian women and 62.5% of US women overestimated the risk of CC (i.e., reported it to be among the top three most common cancers in their country). A higher proportion of Norwegian women (42.2%), compared with U.S. women (32.2%), were able to correctly identify a virus (HPV) as the main cause of CC (Appendix, Table A3).

### 3.3 Information on harms and benefits and stated intentions to participate

**Fig. 2:**
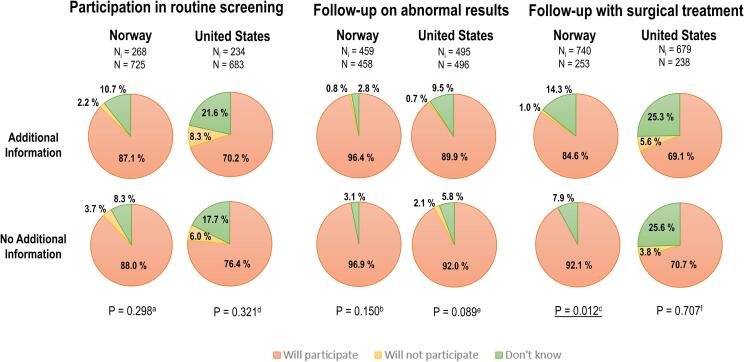
Women’s stated intention to participate in 3 different stages of cervical cancer screening (routine screening, follow-up test and precancer treatment) in both the United States and Norway. Women who had a hysterectomy were excluded from the analysis. Women in each country were collapsed at each stage of the simplified screening into two main groups: women that had received additional information about screening harms and benefits (N_i_), and woman who had not (N). Consequently, the analytic sample size in each of the two collapsed groups changed depending on where in the screening process intention to pursue follow-up and treatment was being measured Post-hoc head-to-head comparison were conducted and should be interpreted as significant when P-value <α/3 = 0.05/3 = 0.0167 (Bonferroni correction). a) Yes vs. No: P-value = 0.268; Yes vs. Don't Know: P-value = 0.302; No vs. Don't know: P-value = 0.135. b) Yes vs. No: P-value = 0.048; Yes vs. Don't Know: P-value = 0.804; No vs. Don't know: P-value = 0.058. c) Yes vs. No: P-value = 0.112; **Yes vs. Don't Know: P-value = 0.012**; No vs. Don't know: P-value = 0.203. d) Yes vs. No: P-value = 0.264; Yes vs. Don't Know: P-value = 0.243; No vs. Don't know: P-value = 0.754. e) Yes vs. No: P-value = 0.055; Yes vs. Don't Know: P-value = 0.168; No vs. Don't know: P-value = 0.016. f) Yes vs. No: P-value = 0.406; Yes vs. Don't Know: P-value = 0.960; No vs. Don't know: P-value = 0.445.

At baseline, with no additional information about harms and benefits, 88% (CI: 85.5–90.2%) of Norwegian women and 76% (CI: 72.3–80.0%) of US women stated they would participate in routine screening. When we provided additional information, intentions to participate in routine screening or follow-up after an abnormal Pap smear were not significantly different in either country (Fig. 2, Tables A4/A5). However, the proportion of women that answered they did not know whether they would seek precancer treatment in Norway significantly increased (from 7.9% to 14.3%; p = 0.012). Responses did not differ between U.S. women who did and did not receive additional information on the risks of precancer treatment (p = 0.707). When evaluated on a Likert scale, the certainty of women’s answer to follow recommendations on treatment was significantly reduced for both the Norwegian and U.S. women (from 9.4 to 9.0 (p < 0.01) and from 8.9 to 8.4 (p < 0.001), respectively (Appendix, Table A4/A5). We found no evidence that receiving additional information more than once increased the likelihood of women stating they did not intent to follow recommendations compared to the other groups.

### 3.4 Women’s information preferences

Receiving additional information was supported in both countries (Table 2), with 87% of Norwegian and 79% of US women reporting that they would like to know that future follow-up test(s) may be required. Support was greatest (>88%) in both countries on being informed of the potential side-effects of the surgical procedures and that, in the absence of treatment, most precancers detected would never develop into cancer.

Preferences for the source of information about CC screening differed substantially between Norwegian and U.S. women (Table 1). The majority (59%) of Norwegian women preferred receiving their information from a national public health agency (i.e., the Cancer Registry of Norway); while the majority (59%) of US women preferred the information came from a specialist care provider. Only 5% of US women preferred to receive information from a public health agency, such as the Center for Disease Control and Prevention.

**Table 1:** Women’s stated preferences for receiving additional information. Women were asked whether they thought the following 3 additional pieces of information should be provided in the letters used during the screening process with the option to answer “Yes”, “No” or “Don’t Know” (DK). Women were then subsequently asked from whom they preferred receiving additional information on the risk and harms related to CC screening from.

**More information about …**			
		USA (N = 1078)	Norway (N = 1058)
The risk of getting an abnormal test result and need for follow-up testing	Yes	78.5%	86.8%
	No or DK	21.5%	13.2%
Potential side effects of the surgical procedure^a^	Yes	88.8%	93.7%
	No or DK	11.2%	6.3%
The risk of overtreatment	Yes	78.5%	84.1%
	No or DK	21.5%	15.9%

**Information preferred to come from…**			

		USA (N = 1078)	Norway (N = 1058)

Public Health Agency*		5.0%	58.8%
General practictionner (GP)		26.3%	22.4%
OB/GYN or other specialist		59.5%	13.0%
I prefer to find info on my own		2.2%	0.1%
I'm not interested in receiving any info		1.5%	0.5%
Other		0.4%	0.5%
Don't know		5.2%	4.7%

*Cancer Registry (Norway), CDC (United States).

^a^Women that received additional information (Groups 2, 3 and 4; N_NOR_ = 784, N_USA_ = 808) received specific information on this topic during the simplified screening process (Block 2), whereas women in the control group did not (Group 1; N_NOR_ = 276, N_USA_ = 276).

**Table 2:** Multivariable logistic regressions for the three questions on intention to participate and follow recommendations at each of the three simplified steps of the screening process. The dependent variable, intention to participate, is coded as 1 = yes, 0 = no or don’t know. Odds ratios are shown as well as the average marginal effect (AME). Women that had a hysterectomy were excluded from the analysis. The income categories are listed in US dollars and were adjusted for purchasing power parity. The ethnicity of Norwegian women was not available in the dataset, therefore the variable “born in Norway” was used as a proxy. All analyses were conducted on the data adjusted with post-stratification weights. **p-value < 0.01, *p-value < 0.05.

	1 *–* Participation in routine screening							
	Norwegian Women				American Women			
Binomial Logistic Regression	Multivariable		Average		Multivariable		Average	
	odds ratio		Margin. Effect		odds ratio		Margin. Effect	
**Additional information received**								
*Additional information received*	1.013		0.11	*	0.766		−4.58	
*No add. information (control)*	*ref.*		*ref.*		*ref.*		*ref.*	
**Income**								
*Less than $24,999*	3.451		11.48		0.739		−4.64	
*$25,000 to $39,999*	0.996		−0.05		0.510		−11.28	
*$40,000 to $59,999*	2.359		8.98		0.551		−9.82	
*$60,000 to $84,999*	1.963		7.48		0.627		−7.46	
*$85,000 to $149,999*	2.515		9.45		0.913		−1.33	
*$150,000 or more*	*ref.*		*ref.*		*ref.*		*ref.*	
**Age**								
*age 20*–*29*	7.044	**	13.55	**	0.865		−2.54	
*age 30*–*39*	2.551	**	8.82		1.266		3.83	
*age 40*–*49*	1.524		4.65		1.029		0.48	
*age 50*–*59*	1.660		5.45		1.042		0.70	
*age 60+*	*ref.*		*ref.*		*ref.*		*ref.*	
**Marital status**								
*Single*	0.998		−0.02		0.635	*	−7.94	*
*Married or Cohabitating*	*ref.*		*ref.*		*ref.*		*ref.*	
**Education**								
*No university or college*	0.945		−0.49		0.675		−6.79	
*University or college*	*ref.*		*ref.*		*ref.*		*ref.*	
**Cause of CC is a virus**								
*HPV*	0.837		−1.53		1.427		5.85	
*incorrect*	*ref.*		*ref.*		*ref.*		*ref.*	
**Ethnicity**								
*African-American/Black*	*–*		*–*		1.238		3.67	
*Hispanic or Latino*	*–*		*–*		1.776		9.13	
*Other*	*–*		*–*		2.310	*	12.50	*
Caucasian	*–*		*–*		*ref*		*ref*	
**Born in Norway**								
*Born in Norway (Yes)*	0.754		−2.20		*–*		*–*	
(No)	*ref*		*ref.*		*–*		*–*	
**Planning future pregnancy**								
*(No or don't know)*	2.198		8.36		0.585		−8.37	
(Yes)	*ref*		*ref.*		*ref*		*ref*	
**Had a previous test before**								
*(No or don't know)*	0.100	**	−37.97	**	0.205	**	−0.33	**
(Yes)	*ref*		*ref.*		*ref*		*ref*	

	2 – Follow-up on abnormal results (control Pap-smear)							
	Norwegian Women				American Women			
Binomial Logistic Regression	Multivariable		Average		Multivariable		Average	
	odds ratio		Margin. Effect		odds ratio		Margin. Effect	
**Additional information received**								
*Additional information received*	0.841		−0.50		0.834		−1.21	
*No add. information (control)*	*ref.*		*ref*		*ref.*		*ref*	
**Income**								
*Less than $24,999*	0.649		−1.73		0.085	**	−12.53	**
*$25,000 to $39,999*	0.452		−3.73		0.141	*	−8.07	*
*$40,000 to $59,999*	0.996		2.17		0.091	**	−11.89	**
*$60,000 to $84,999*	2.662		0.92		0.198		−5.76	*
*$85,000 to $149,999*	1.377				0.337			
*$150,000 or more*	*ref.*		*ref*		*ref.*		*ref*	
**Age**								
*age 20*–*29*	2.531		2.22		0.605		−4.65	
*age 30*–*39*	0.930		−0.26		1.620		3.32	
*age 40*–*49*	1.414		1.04		1.142		1.02	
*age 50*–*59*	1.601		1.34		4.549	**	7.56	*
*age 60+*	*ref.*		*ref*		*ref.*		*ref*	
**Marital status**								
*Single*	0.928		−0.22		2.451	*	5.58	**
*Married or Cohabitating*	*ref.*		*ref*		*ref.*		*ref*	
**Education**								
*No university or college*	0.559		−1.72		0.534		−4.33	
*University or college*	*ref.*		*ref*		*ref.*		*ref*	
**Cause of CC is a virus**								
*HPV*	0.9576		−0.13		1.823		3.72	
*incorrect*	*ref.*		*ref*		*ref.*		*ref*	
**Ethnicity**								
*African-American/Black*	*–*		*–*		0.427		−6.80	
*Hispanic or Latino*	*–*		*–*		2.106		3.59	
*Other*	*–*		*–*		0.273	*	−11.82	
Caucasian	*–*		*–*		*ref*		*ref*	
**Born in Norway**								
*Born in Norway (Yes)*	2.602		3.98		*–*		*–*	
(No)	*ref*		*ref*		*–*		*–*	
**Planning future pregnancy**								
*(No or don't know)*	0.984		−0.05		0.647		−2.70	
(Yes)	*ref*		*ref*		*ref*		*ref*	
**Had a previous test before**								
*(No or don't know)*	0.118	**	−14.18		0.160	**	−19.36	*
(Yes)	*ref*		*ref*		*ref*		*ref*	

	3 – Follow-up with surgical treatment							
	Norwegian Women				American Women			
Binomial Logistic Regression	Multivariable		Average		Multivariable		Average	
	odds ratio		Margin. Effect		odds ratio		Margin. Effect	
**Additional information received**								
*Additional information received*	0.373	**	−8.26	**	0.955		−0.87	
*No add. information (control)*	*ref.*		*ref*		*ref.*		*ref*	
**Income**								
*Less than $24,999*	0.499		−6.46		0.480		−12.80	
*$25,000 to $39,999*	0.455		−7.57		0.379	*	−17.73	*
*$40,000 to $59,999*	0.459		−7.46		0.443		−14.44	*
*$60,000 to $84,999*	0.585		−4.68		0.462		−13.61	
*$85,000 to $149,999*	0.797				0.657			
*$150,000 or more*	*ref.*		*ref*		*ref.*		*ref*	
**Age**								
*age 20*–*29*	1.961		7.28		0.495		−13.25	
*age 30*–*39*	1.208		2.39		0.518	*	−12.29	*
*age 40*–*49*	1.811		6.59		0.883		−2.08	
*age 50*–*59*	2.197		8.19		0.724		−5.65	
*age 60+*	*ref.*		*ref*		*ref.*		*ref*	
**Marital status**								
*Single*	1.046		0.46		0.902		−1.98	
*Married or Cohabitating*	*ref.*		*ref*		*ref.*		*ref*	
**Education**								
*No university or college*	0.773		−2.67		0.740		−5.90	
*University or college*	*ref.*		*ref*		*ref.*		*ref*	
**Cause of CC is a virus**								
*HPV*	1.064		0.63		1.872	**	11.75	**
*incorrect*	*ref.*		*ref*		*ref.*		*ref*	
**Ethnicity**								
*African-American/Black*	*–*		*–*		0.676		−7.87	
*Hispanic or Latino*	*–*		*–*		0.672		−8.02	
*Other*	*–*		*–*		1.431		6.29	
Caucasian	*–*		*ref*		*ref*		*ref*	
**Born in Norway**								
*Born in Norway (Yes)*	1.111		–		*–*		*–*	
(No)	*ref*		*ref*		*–*		*–*	
**Planning future pregnancy**								
*(No or don't know)*	1.682		6.03		0.986		−0.26	
(Yes)	*ref*		*ref*		*ref*		*ref*	
**Had a previous test before**								
*(No or don't know)*	0.426	*	−11.30		0.452	*	−16.88	
(Yes)	*ref*		*ref*		*ref*		*ref*	

### 3.5 Socioeconomic and other factors and stated intentions to participate

In both countries, the most important predictor of stated intention to participate in routine screening was history of a prior Pap test (Table 2; right panel). The probabilities of attending screening were 38.0 and 0.33 percentage points (PP) (p < 0.01) lower among Norwegian and U.S. women who never had a Pap test before. In addition, compared to women aged >60 years, Norwegian women aged 20–29 were 13.6PP (p < 0.01) more likely to respond that they would participate in screening. Age-specific differences in intention to screen were not detected among U.S. respondents. Single U.S. women were 7.9PP less likely (p < 0.05) to attend screening comared with women who were married/cohabitating. In addition, U.S. women that reported being of ‘other’ ethnicity had a 12.5PP (p < 0.05) higher probability of intending to attend screening compared with Caucasian respondents. No similar trends were detected among Norwegian women.

Having a prior Pap test was also the most important predictor of stated intention to follow-up recommendations in both countries (Table 2; middle panel). U.S. women were significantly less likely to follow-up on abnormal results if their household income was lower than $85,000 compared with earning >$150,000. However, they were more likely to adhere to recommendations if they were single. Similar income and civil status effects were not detected among Norwegian women.

Having a prior Pap test was significantly associated with lower stated intentions to adhere to precancer treatment in both countries (Table 2; right panel). In addition, women with lower household income and of younger age had a lower probability of intending to seek treatment in the U.S. In Norway, income trends were not statistically significant. Finally, US women who had baseline knowledge of the causal factor of CC had a higher probability of 11.8PP (p < 0.01) to seek treatment compared with women that did not know.

## 4 Discussion

We found that providing additional information did not cause a significant shift in women’s intentions to participate in routine screening or follow-up recommendations in either country; although additional information resulted in more Norwegian women responding that they did not know if they would seek treatment. A similar shift in uncertainty was found among U.S. women when measured on a Likert-scale. Given the simplified letters only used the term “surgical procedure”, women may have made assumptions about the degree of invasiveness of these procedures and became uncertain. In reality, women may have an open dialogue with their physician to gain clarity before making a decision. Our findings suggest that risk information, while favored to promote a patient’s right and ability to make decisions about themselves, may cause hesitation without necessarily changing final choice. Nevertheless, the increased uncertainty may indicate that women absorbed the information provided and may require help to make a more critical assessment before deciding. Skinner et al. (2016) showed that more tailored information about colorectal cancer prompted patients to increase their discussions about risk and screening with their physicians. Therefore, more complete information in letters may not impact participation, but could potentially impact clinical practice.

While women in both countries were overwhelmingly in favor of receiving additional information on harms related to CC screening, they differed in their source preferences. U.S. women preferred it came directly from their specialist or obstetrician/gynecologist (OB/GYN) while Norwegian women preferred receiving it from the National Cancer Registry. These findings are consistent with studies showing that Norwegian women have a higher level of trust in public authorities and prefer information from organized programs informed by experts (Østerlie et al., 2008). This may reflect that in the U.S. trust in public authorities is lower than in specialists and that they also prefer information coming from their most trusted source. It is important to note that for many women, a primary care provider was their preferred source.

Exploring factors that are known contributors to changes in intentions to participate in screening, we found that having taken a Pap test in the past was important in both countries, a finding previously reported in the U.K. (Labeit et al., 2013). Generally, apart from age, none of the factors we explored predicted Norwegian women’s intention at any stage of screening. In contrast, among US women, the lowest income groups were more likely to state they would not follow-up after abnormal tests or seek precancer treatment compared to the highest income group. This is consistent with previous studies that found a lower participation rate for women with lower socio-economic backgrounds (Johnson et al., 2008). Lower household income may represent a barrier to participate in screening due to a lower ability to pay for tests and procedures. Considering that lower income or unemployment is associated with not having health insurance in the U.S. (Barnett and Berchick, 2017), understanding how the ability to pay impacts screening participation is particularly relevant for policymakers. With the introduction of the Affordable Care Act (ACA) in 2014, insurers were required to provide a variety of preventive services to women, such as cervical Pap smears, at no cost. However, as the ACA is continuously being challenged, pre-instituting potential income barriers may negatively impact participation once again. As our survey was conducted prior to the ACA’s implementation, women’s participation while in effect may have since changed or increased.

### 4.1 Limitations

While our survey was a good proxy for the Norwegian context where invitations by the Cancer Registry is routine, adapting these letters to fit the U.S. context may limit the generalizability of our results to the U.S. setting. While 62% of respondents in a recent study reported trusting the CDC (Kowitt et al., 2017), it is possible that U.S. women would not expect to receive invitation letters for CC screening directly from them. The fact that invitation letters are familiar for Norwegian women, but not for U.S. women, could explain in part why we measured a higher degree of skepticism in U.S. women. This hypothesis is also supported by our findings that the CDC was not their preferred source of information.

While exploring the impact of socioeconomic and other factors, we recoded responses “don’t know” and “no” together, to enable us conducting binomial logistic regressions. This grouping was motivated by the fact that the goal of invitation letters is to increase participation. Answering “don’t know” does not secure participation. We do not know whether women expressing uncertainty would have ultimately attended screening and followed-up. These answers might represent women who need more time to process the information before deciding to participate or require a discussion of trade-offs more thoroughly with their physician before consenting.

While the findings of our regression models seemed to align with previous research, it is important to note that our measured endpoint is intention to participate. Our results are subject to hypothetical bias; we did not measure actual behavior. However, previous research supports that an intention to perform an action is predictive of actual behavior (Ajzen, 1991) and it was previously demonstrated that providing additional information on harms and benefits influenced screening behavior (Hestbech et al., 2016, Perneger et al., 2011, Adab et al., 2003).

Finally, it is difficult to assess whether women responding to our survey read the information letters thoroughly, including the additional information provided. If they did not, then women in the additional information group may not have processed the information as assumed in our analysis.

## 5 Conclusions

Our findings suggest that providing more balanced information on harms and benefits may not impact women’s intentions to participate in CC screening. Nevertheless, we observe increased uncertainty regarding decisions to seek precancer treatment, especially in Norway. Despite so, women in both countries strongly supported receiving additional information on harms and benefits, but they had strong system-specific preferences for the sources of information. While socio-economic factors helped predict trends in intention to participate for U.S. women, the same was not found for Norwegian women. If re-designing and improving letters aims to increase participation, careful consideration should be given to country-context and socioeconomic barriers to screening. Unbiased information improves on the ethical principle of respect for autonomy and self-determination but gaining a deeper understanding of how providing additional information impacts understanding of risk involved with CC screening and decision-making may be important for programs that aim to improve the quality of women’s decisions.

## Source of funding

University of Oslo.

## CRediT authorship contribution statement

**P.R. Cyr:** Conceptualization, Methodology, Software, Formal analysis, Writing - original draft, Writing - review & editing, Visualization. **K. Pedersen:** Conceptualization, Methodology, Writing - original draft, Writing - review & editing. **A.L. Iyer:** Conceptualization, Methodology, Investigation, Writing - review & editing. **M.K. Bundorf:** Conceptualization, Project administration, Writing - review & editing. **J.D. Goldhaber-Fiebert:** Conceptualization, Methodology, Investigation, Writing - review & editing. **D. Gyrd-Hansen:** Conceptualization, Writing - review & editing. **I.S. Kristiansen:** Conceptualization, Writing - review & editing, Project administration, Funding acquisition, Supervision. **E.A. Burger:** Conceptualization, Methodology, Writing - original draft, Writing - review & editing, Visualization, Supervision.

## Declaration of Competing Interest

The authors declare that they have no known competing financial interests or personal relationships that could have appeared to influence the work reported in this paper.
