# Supplementary material for: Providing more balanced information on the harms and benefits of cervical cancer screening: A randomized survey among US and Norwegian women
Source: Prev Med Rep. 2021 Jun 23;23:101452. doi: 10.1016/j.pmedr.2021.101452 (PMC8242055; doi:10.1016/j.pmedr.2021.101452)
Supplement: Supplementary data 1 [file mmc1.docx]

**Supplementary Appendix for the accompanying manuscript:**

Information on the Harms and Benefits of Cervical Cancer Screening: A Randomized Survey Among US and Norwegian Women

**Figure A1: Flow diagram of recruitment and randomization**


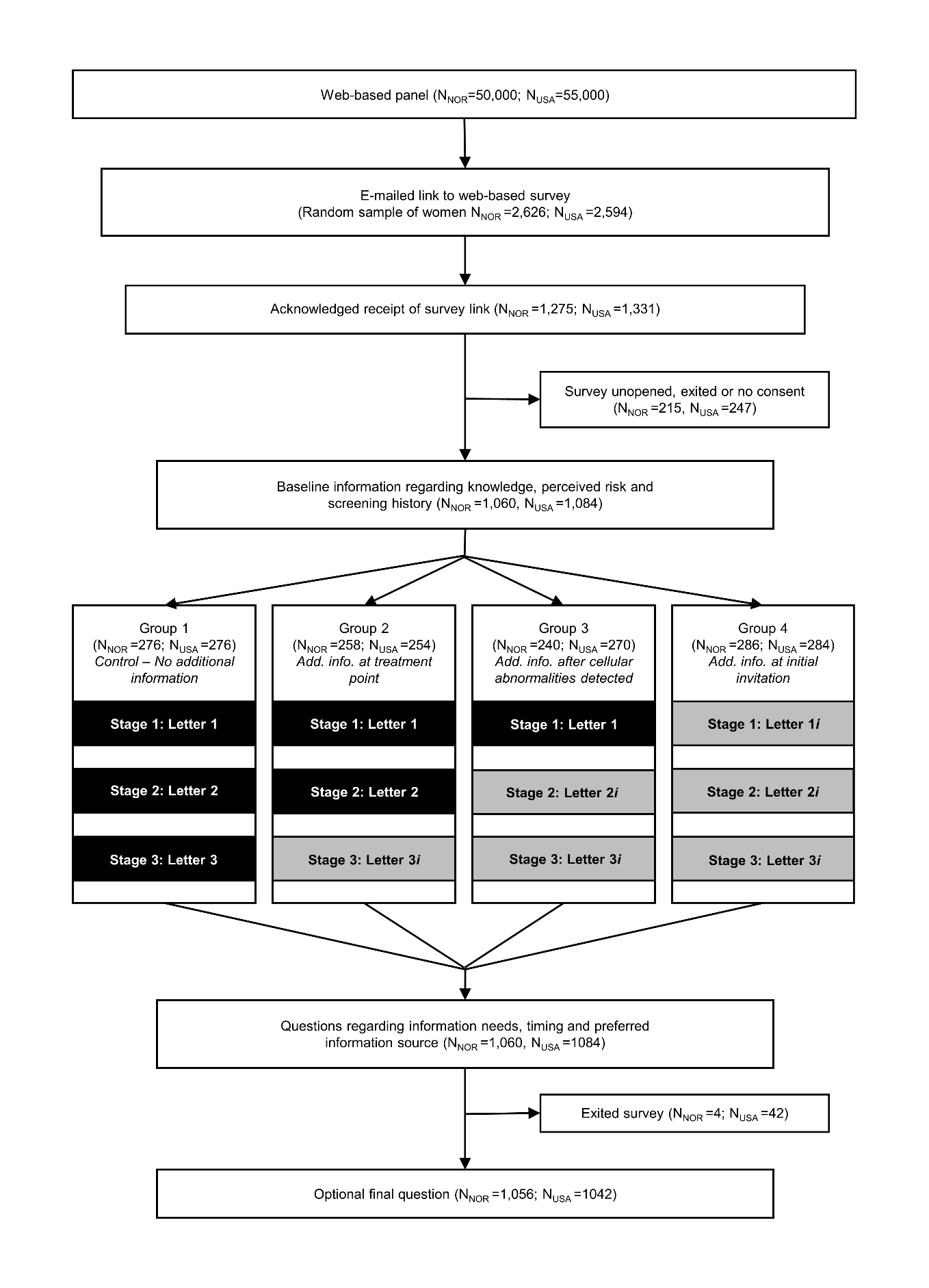


**Sample of U.S. Women**

The GfK group conducted the survey with its KnowledgePanel which is a pre-recruited representative sample of approximately 55,000 individuals from the U.S. population. Panel members are randomly recruited through probability-based sampling, and households with no computers or internet access were told that, as reward for completing a short survey weekly, they would be provided with free monthly Internet access and a laptop computer. Incentive points per survey, redeemable for cash, are usually given to respondents for completing their surveys, but these same rewards are not given to panel members provided with a laptop computer and free Internet. However, no payments were given for participation in this study.

Our survey targeted a nationally representative sample of U.S. adult women, ages 21-65, which was selected at random from the panel (see distribution of characteristics in **Table A1a**). Study-specific post-stratification weights were constructed by Gfk so that the data can be adjusted for non-responses, over- and under-sampling, and non-coverage. Demographic and geographic distributions are used as benchmarks in this adjustment. They include age, ethnicity, education level, household income, census region (Northeast, Midwest, South, West), metropolitan area (yes/no), internet access (yes/no).

The distribution of baseline characteristics of American women after we applied the weights are reported in **Table A1b** and showed good balance across the four randomized groups.

**Table A1a**: Baseline characteristics of surveyed US women (raw and unweighted data).

|  | **Group 1** | | **Group 2** | | **Group 3** | | **Group 4** | |  |  |
| --- | --- | --- | --- | --- | --- | --- | --- | --- | --- | --- |
| **Variables** | No additional info (control) | | Additional info at treatment | | Additional info at abnormal results | | Additional info at initial invitation | | **Total** | |
|  |  | |  | |  | |  | |  | |
|  | **N=276** | | **N=254** | | **N=270** | | **N=284** | | **N=1084** | |
| **Age (years), mean (SE)** | **45.56** | (.74) | **45.49** | (.85) | **44.87** | (.76) | **44.69** | (.78) | **45.14** | (.39) |
|  |  |  |  |  |  |  |  |  |  |  |
| **Age groups** | **N=276** | | **N=254** | | **N=270** | | **N=284** | | **N=1084** | |
| <30 | **36** | 13.0 % | **41** | 16.1 % | **41** | 15.2 % | **53** | 18.7 % | **171** | 15.8 % |
| 30-39 | **59** | 21.4 % | **50** | 19.7 % | **55** | 20.4 % | **48** | 16.9 % | **212** | 19.6 % |
| 40-49 | **64** | 23.2 % | **44** | 17.3 % | **64** | 23.7 % | **65** | 22.9 % | **237** | 21.9 % |
| 50-59 | **72** | 26.1 % | **73** | 28.7 % | **67** | 24.8 % | **75** | 26.4 % | **287** | 26.5 % |
| 60+ | **45** | 16.3 % | **46** | 18.1 % | **43** | 15.9 % | **43** | 15.1 % | **177** | 16.3 % |
|  |  |  |  |  |  |  |  |  |  |  |
| **Household annual income (USD)** | **N=276** | | **N=254** | | **N=270** | | **N=284** | | **N=1084** | |
| Less than $24,999 | **41** | 14.9 % | **46** | 18.1 % | **45** | 16.7 % | **49** | 17.3 % | **181** | 16.7% |
| $25,000 to $39,999 | **39** | 14.1 % | **36** | 14.2 % | **37** | 13.7 % | **41** | 14.4 % | **153** | 14.1% |
| $40,000 to $59,999 | **44** | 15.9 % | **26** | 10.2 % | **46** | 17.0 % | **52** | 18.3 % | **168** | 15.5% |
| $60,000 to $84,999 | **52** | 18.8 % | **47** | 18.5 % | **50** | 18.5 % | **49** | 17.3 % | **198** | 18.3% |
| $85,000 to $149,999 | **70** | 25.4 % | **78** | 30.7 % | **61** | 22.6 % | **74** | 26.1 % | **283** | 26.1% |
| $150,000 or more | **30** | 10.9 % | **21** | 8.3 % | **31** | 11.5 % | **19** | 6.7 % | **101** | 9.3% |
|  |  |  |  |  |  |  |  |  |  |  |
|  |  |  |  |  |  |  |  |  |  |  |
| **Geographic location** | **N=276** | | **N=254** | | **N=270** | | **N=284** | | **N=1084** | |
| Northeast | **49** | 17.8 % | **48** | 18.9 % | **47** | 17.4 % | **49** | 17.3 % | **193** | 17.8 % |
| Midwest | **55** | 19.9 % | **62** | 24.4 % | **63** | 23.3 % | **78** | 27.5 % | **258** | 23.8 % |
| South | **101** | 36.6 % | **89** | 35.0 % | **96** | 35.6 % | **107** | 37.7 % | **393** | 36.3 % |
| West | **71** | 25.7 % | **55** | 21.7 % | **64** | 23.7 % | **50** | 17.6 % | **240** | 22.1 % |
|  |  |  |  |  |  |  |  |  |  |  |
| **Marital status** | **N=276** | | **N=254** | | **N=270** | | **N=284** | | **N=1084** | |
| Married/cohabitating | **202** | 73.2 % | **165** | 65.0 % | **170** | 63.0 % | **191** | 67.3 % | **728** | 67.2 % |
| Single | **74** | 26.8 % | **89** | 35.0 % | **100** | 37.0 % | **93** | 32.8 % | **356** | 32.8 % |
|  |  |  |  |  |  |  |  |  |  |  |
|  |  |  |  |  |  |  |  |  |  |  |
| **Education** | **N=276** | | **N=254** | | **N=270** | | **N=284** | | **N=1084** | |
| ≤High school | **92** | 33.3 % | **87** | 34.3 % | **91** | 33.7 % | **98** | 34.5 % | **368** | 34.0 % |
| ≥1 year of university or college | **184** | 66.7 % | **167** | 65.8 % | **179** | 66.3 % | **186** | 65.5 % | **716** | 66.1 % |
|  |  |  |  |  |  |  |  |  |  |  |
| **Ethnicity** | **N=276** | | **N=254** | | **N=270** | | **N=284** | | **N=1084** | |
| Caucasian | **196** | 71.0 % | **189** | 74.4 % | **201** | 74.4 % | **206** | 72.5 % | **792** | 73.1 % |
| African-American/Black | **28** | 10.1 % | **31** | 12.2 % | **23** | 8.5 % | **34** | 12.0 % | **116** | 10.7 % |
| Hispanic or Latino | **30** | 10.9 % | **23** | 9.1 % | **26** | 9.6 % | **25** | 8.8 % | **104** | 9.6 % |
| Other | **22** | 10.0 % | **11** | 4.3 % | **20** | 7.4 % | **19** | 6.7 % | **72** | 6.6 % |
|  |  |  |  |  |  |  |  |  |  |  |
| **Ever had a Pap test done?** | **N=276** | | **N=254** | | **N=270** | | **N=284** | | **N=1084** | |
| Yes | **260** | 94.2 % | **245** | 96.5 % | **252** | 93.3 % | **268** | 94.4 % | **1025** | 94.6 % |
| No | **8** | 2.9 % | **9** | 3.5 % | **11** | 4.1 % | **12** | 4.2 % | **40** | 3.7 % |
| Don't know | **2** | 0.7 % | **0** | 0.0 % | **5** | 1.9 % | **3** | 1.1 % | **10** | 0.9 % |
| *(missing / did not answer)* | **6** | 2.2 % | **0** | 0.0 % | **2** | 0.7 % | **1.0** | 0.4 % | **9** | 0.8 % |
|  |  |  |  |  |  |  |  |  |  |  |
| **Hysterectomized** | **N=276** | | **N=254** | | **N=270** | | **N=284** | | **N=1084** | |
| Yes | **37** | 13.4 % | **33** | 13.0 % | **45** | 16.7 % | **49** | 17.3 % | **164** | 15.8 % |
| No | **234** | 84.8 % | **216** | 85.0 % | **220** | 81.5 % | **230** | 81.0 % | **900** | 83.0 % |
| Don't know | **2** | 0.7 % | **2** | 0.8 % | **5** | 1.9 % | **4** | 1.4 % | **13** | 1.2 % |
| *(missing / did not answer)* | **3** | 1.1 % | **3** | 1.2 % | **0** | 0.0 % | **1** | 0.4 % | **7** | 0.7 % |
|  |  |  |  |  |  |  |  |  |  |  |
| **Planning future pregnancy?** | **N=276** | | **N=254** | | **N=270** | | **N=284** | | **N=1084** | |
| Yes | **43** | 15.6 % | **30** | 11.8 % | **42** | 15.6 % | **41** | 14.4 % | **156** | 14.4 % |
| No | **215** | 77.9 % | **207** | 81.5 % | **212** | 78.5 % | **222** | 78.2 % | **856** | 79.0 % |
| Don't know | **15** | 5.4 % | **16** | 6.3 % | **15** | 5.6 % | **19** | 6.7 % | **65** | 6.0 % |
| *(missing / did not answer)* | **3** | 1.1 % | **1** | 0.4 % | **1** | 0.4 % | **2** | 0.7 % | **7** | 0.7 % |

**Table A1b**: Baseline characteristics of surveyed US women adjusted by post-stratification weight.

|  | **Group 1** | | **Group 2** | | **Group 3** | | **Group 4** | |  |  |
| --- | --- | --- | --- | --- | --- | --- | --- | --- | --- | --- |
| **Variables** | No additional info (control) | | Additional info at treatment | | Additional info at abnormal results | | Additional info at initial invitation | | **Total** | |
|  |  | |  | |  | |  | |  | |
|  | **N=276** | | **N=255** | | **N=270** | | **N=283** | | **N=1084** | |
| **Age (years), mean (SD)** | 42.94 | (12,6) | 43.06 | (13,3) | 43.3 | (12,7) | 43.0 | (13,1) | 43.07 | (12,9) |
|  |  |  |  |  |  |  |  |  |  |  |
| **Age groups** | **N=276** | | **N=254** | | **N=270** | | **N=284** | | **N=1084** | |
| <30 | 56.4 | 20.4 % | 51.8 | 20.4 % | 55.3 | 20.5 % | 58.4 | 20.6 % | 221.8 | 20.5 % |
| 30-39 | 66.6 | 24.2 % | 58.6 | 23.1 % | 51.5 | 19.1 % | 62.4 | 22.0 % | 239.1 | 22.1 % |
| 40-49 | 55.9 | 20.3 % | 43.4 | 17.1 % | 63.7 | 23.6 % | 58.6 | 20.6 % | 221.7 | 20.5 % |
| 50-59 | 63.9 | 23.2 % | 69.7 | 27.4 % | 67.0 | 24.8 % | 70.3 | 24.7 % | 270.9 | 25.0 % |
| 60-69 | 33.1 | 12.0 % | 30.6 | 12.0 % | 32.5 | 12.0 % | 34.3 | 12.1 % | 130.5 | 12.0 % |
|  |  |  |  |  |  |  |  |  |  |  |
| **Household annual income (USD)** | **N=276** | | **N=254** | | **N=270** | | **N=284** | | **N=1084** | |
| Less than $24,999 | 49.1 | 17.8 % | 45.4 | 17.9 % | 47.8 | 17.7 % | 50.4 | 17.8 % | 192.7 | 18% |
| $25,000 to $39,999 | 44.0 | 15.9 % | 41.9 | 16.5 % | 33.1 | 12.3 % | 44.2 | 15.6 % | 163.1 | 15% |
| $40,000 to $59,999 | 41.6 | 15.1 % | 31.6 | 12.5 % | 47.8 | 17.7 % | 39.9 | 14.1 % | 160.9 | 15% |
| $60,000 to $84,999 | 36.0 | 13.0 % | 51.4 | 20.2 % | 51.9 | 19.2 % | 44.0 | 15.5 % | 183.3 | 17% |
| $85,000 to $149,999 | 71.1 | 25.7 % | 65.3 | 25.7 % | 59.4 | 22.0 % | 82.9 | 29.2 % | 278.6 | 26% |
| $150,000 or more | 34.3 | 12.4 % | 18.4 | 7.2 % | 30.0 | 11.1 % | 22.7 | 8.0 % | 105.4 | 10% |
|  |  |  |  |  |  |  |  |  |  |  |
| **Geographic location** | **N=276** | | **N=254** | | **N=270** | | **N=284** | | **N=1084** | |
| Northeast | 49.7 | 18.0 % | 46.5 | 18.3 % | 49.4 | 18.3 % | 51.6 | 18.2 % | 197.2 | 18.2 % |
| Midwest | 58.4 | 21.2 % | 54.5 | 21.5 % | 57.5 | 21.3 % | 61.2 | 21.5 % | 231.6 | 21.4 % |
| South | 103.6 | 37.5 % | 93.6 | 36.9 % | 101.4 | 37.6 % | 106.7 | 37.6 % | 405.3 | 37.4 % |
| West | 64.3 | 23.3 % | 59.4 | 23.4 % | 61.7 | 22.9 % | 64.5 | 22.7 % | 249.9 | 23.1 % |
|  |  |  |  |  |  |  |  |  |  |  |
| **Marital status** | **N=276** | | **N=254** | | **N=270** | | **N=284** | | **N=1084** | |
| Married/cohabitating | 203.4 | 73.7 % | 160.2 | 63.1 % | 168.3 | 62.3 % | 191.7 | 67.5 % | 723.6 | 66.8 % |
| Single | 72.7 | 26.3 % | 93.8 | 36.9 % | 101.7 | 37.7 % | 92.3 | 32.5 % | 360.4 | 33.3 % |
|  |  |  |  |  |  |  |  |  |  |  |
| **Education** | **N=276** | | **N=254** | | **N=270** | | **N=284** | | **N=1084** | |
| ≤High school | 99.26 | 36.0 % | 90.85 | 35.8 % | 97.37 | 36.1 % | 102.8 | 36.2 % | 390.3 | 36.0 % |
| ≥1 year of university or college | 176.7 | 64.0 % | 163.2 | 64.2 % | 172.6 | 63.9 % | 181.2 | 63.8 % | 693.7 | 64.0 % |
|  |  |  |  |  |  |  |  |  |  |  |
| **Ethnicity** | **N=276** | | **N=254** | | **N=270** | | **N=284** | | **N=1084** | |
| Caucasian | 169.7 | 61.5 % | 168.8 | 66.5 % | 169.2 | 62.7 % | 181.9 | 64.1 % | 689.5 | 63.6 % |
| African-American/Black | 35.5 | 12.9 % | 32.8 | 12.9 % | 34.8 | 12.9 % | 36.6 | 12.9 % | 139.6 | 12.9 % |
| Hispanic or Latino | 42.9 | 15.6 % | 38.8 | 15.3 % | 41.6 | 15.4 % | 42.2 | 14.9 % | 165.5 | 15.3 % |
| Other | 27.9 | 10.1 % | 13.7 | 5.4 % | 24.5 | 9.1 % | 23.2 | 8.2 % | 89.3 | 8.2 % |
|  |  |  |  |  |  |  |  |  |  |  |
| **Ever had a Pap test done?** | **N=270,2** | | **N=254** | | **N=268,6** | | **N=282,7** | | **N=1075** | |
| Yes | 251.8 | 93.2 % | 244.1 | 96.1 % | 250.1 | 93.1 % | 260.9 | 92.3 % | 1007.0 | 93.6 % |
| No | 11.2 | 4.2 % | 9.9 | 3.9 % | 14.1 | 5.3 % | 16.4 | 5.8 % | 51.6 | 4.8 % |
| Don't know | 7.1 | 2.6 % | 0.0 | 0.0 % | 4.375 | 1.6 % | 5.5 | 1.9 % | 16.96 | 1.6 % |
|  |  |  |  |  |  |  |  |  |  |  |
| **Hysterectomized** | **N=273** | | **N=250,5** | | **N=270** | | **N=282,7** | | **N=1076** | |
| Yes | 29.9 | 11.0 % | 29.0 | 11.5 % | 42.4 | 15.7 % | 42.8 | 15.1 % | 144.0 | 15.2 % |
| No | 238.8 | 87.5 % | 221.4 | 88.4 % | 218.2 | 80.8 % | 236.9 | 83.8 % | 915.3 | 83.6 % |
| Don't know | 4.3 | 1.6 % | 0.2 | 0.1 % | 9.4 | 3.5 % | 3.0 | 1.1 % | 17.0 | 1.2 % |
|  |  |  |  |  |  |  |  |  |  |  |
| **Planning future pregnancy?** | **N=274,2** | | **N=253,4** | | **N=269,5** | | **N=282,3** | | **N=1079** | |
| Yes | 63.9 | 23.3 % | 34.7 | 13.7 % | 52.5 | 19.5 % | 41.4 | 14.6 % | 192.4 | 17.8 % |
| No | 190.4 | 69.4 % | 201.4 | 79.5 % | 202.8 | 75.3 % | 216.6 | 76.7 % | 811.2 | 75.2 % |
| Don't know | 20.0 | 7.3 % | 17.3 | 6.8 % | 14.2 | 5.3 % | 24.4 | 8.6 % | 75.8 | 7.0 % |

**Table A2c**: Baseline characteristics of surveyed US women adjusted by post-stratification weight compared to the US population distribution

|  |  |  |  |  |  |
| --- | --- | --- | --- | --- | --- |
|  | **USA Survey Population** | | | **USA Population*** | |
|  |  |  |  |  |  |
|  | **Age groups** | **N=1084** | |  | **Age groups** |
|  | <30 | 221.8 | 20.5 % | 21.1% | <30 |
|  | 30-39 | 239.1 | 22.1 % | 19.8 % | 30-39 |
|  | 40-49 | 221.7 | 20.5 % | 20.8 % | 40-49 |
|  | 50-59 | 270.9 | 25.0 % | 21.8 % | 50-59 |
|  | 60-69 | 130.5 | 12.0 % | 16.6 % | 60-69 |
|  |  |  |  |  |  |
|  | **Household annual income (USD)** | **N=1084** | |  | **Household annual income (USD)^a^** |
|  | Less than $24,999 | 192.7 | 18% | 21.4% | under $24,999 |
|  | $25,000 to $39,999 | 163.1 | 15% | 23.0% | $25,000 to $49,999 |
|  | $40,000 to $59,999 | 160.9 | 15% | 18.4% | $50,000 to $74,999 |
|  | $60,000 to $84,999 | 183.3 | 17% | 12.7% | $75,000 to $99,999 |
|  | $85,000 to $149,999 | 278.6 | 26% | 24.5% | $100,000 and over |
|  | $150,000 or more | 105.4 | 10% | - |  |
|  |  |  |  |  |  |
|  | **Geographic location** | **N=1084** | |  | **Geographic location^b^** |
|  | Northeast | 197.2 | 18.2 % | 18.1 % | Northeast |
|  | Midwest | 231.6 | 21.4 % | 21.7 % | Midwest |
|  | South | 405.3 | 37.4 % | 37.2 % | South |
|  | West | 249.9 | 23.1 % | 23.0 % | West |
|  |  |  |  |  |  |
|  | **Marital status** | **N=1084** | |  | **Marital status** |
|  | Married/cohabitating | 723.6 | 66.8 % | 53.0 % | Married / excluding cohab. |
|  | Single | 360.4 | 33.3 % | - | - |
|  |  |  |  |  |  |
|  | **Education** | **N=1084** | |  | **Education** |
|  | ≤High school | 390.3 | 36.0 % | 37.6 % | ≤High school |
|  | ≥1 year of university or college | 693.7 | 64.0 % | 62.4 % | ≥1 year of university or college |
|  |  |  |  |  |  |
|  | **Ethnicity** | **N=1084** | |  | **Ethnicity** |
|  | Caucasian | 689.5 | 63.6 % | 62.5 % | Caucasian |
|  | African-American/Black | 139.6 | 12.9 % | 12.7 % | African-American/Black |
|  | Hispanic or Latino | 165.5 | 15.3 % | 16.6 % | Hispanic or Latino |
|  | Other | 89.3 | 8.2 % | 8.2 % | Other |
|  |  |  |  |  |  |
| * | Data from the United States Census Bureau | | |  |  |
| ^a^ | Household income table was not available with same categorical cut-offs we used | | | | |
| ^b^ | Data from 2010 USA Census |  |  |  |  |

**Sample of Norwegian Women**

The TNS Gallup access panel is a pre-recruited sample of approximately 50,000 people over the age of 15 willing to participate in surveys. Participants are recruited at random through telephone and postal surveys. The panel participants characteristics have already been mapped. The survey sample selection was therefore pre-stratified by age, gender, place of residence and level of education, and drawn at random within the strata.

Participants to this study were recruited with an e-mail invitation. Internet access is high among all age groups in Norway (over 95%) but is slightly lower in the age group of 60-66 (86%) but was deemed to be adequate for the survey’s target group (women aged between 25 to 69). Women were not rewarded for participation in the survey.

The final sample showed a good spread over the age and region categories (see **Table A2a**), but the 50–59-year-old were slightly overrepresented. Women who attended at least one year of college or university were slightly over-sampled when compared to the Norwegian population. While according to Table A2c, it seems that women in the lowest personal income bracket group have been slightly under-sampled, we instead used household income in our regression. While direct comparison is not possible (census data on households from 2013 not available), we expect the number of households in the lowest income bracket to be very small.

Given that the pre-stratification was done for recruitment to the survey, but not for the randomization into the four groups, it is importance to ensure they are balanced. Post-stratification weights were constructed by Gallup based on age, place of residence and education. The distribution of baseline characteristics of Norwegian women after we applied the weights had improved balance across the groups and is reported in **Table A2b**.

**Table** **A2a**: Baseline characteristics of surveyed Norwegian women (raw and unweighted data)

|  | **Group 1** | | **Group 2** | | **Group 3** | | **Group 4** | |  |  |
| --- | --- | --- | --- | --- | --- | --- | --- | --- | --- | --- |
| **Variables** | No additional info (control) | | Additional info at treatment | | Additional info at abnormal results | | Additional info at initial invitation | | **Total** | |
|  |  | |  | |  | |  | |  | |
|  | **N=276** | | **N=258** | | **N=240** | | **N=286** | | **N=1060** | |
| **Age (years), mean (SE)** | **45.79** | (.65) | **46.81** | (.70) | **46.35** | (.69) | **45.38** | (.65) | **46.05** | (.33) |
|  |  |  |  |  |  |  |  |  |  |  |
| **Age groups** | **N=272,7** | | **N=265,2** | | **N=240.2** | | **N=286** | | **N=1060** | |
| <30 | **11** | 4.0 % | **13** | 5.0 % | **12** | 5.0 % | **19** | 6.6 % | **55** | 5.2 % |
| 30-39 | **73** | 26.5 % | **61** | 23.6 % | **56** | 23.3 % | **79** | 27.6 % | **269** | 25.4 % |
| 40-49 | **87** | 31.5 % | **73** | 28.3 % | **75** | 31.3 % | **73** | 25.5 % | **308** | 29.1 % |
| 50-59 | **81** | 29.4 % | **75** | 29.1 % | **70** | 29.2 % | **95** | 33.2 % | **321** | 30.3 % |
| 60+ | **24** | 8.7 % | **36** | 14.0 % | **27** | 11.3 % | **20** | 7.0 % | **107** | 10.1 % |
|  |  |  |  |  |  |  |  |  |  |  |
| **Household annual income (NOK)** | **N=276** | | **N=258** | | **N=240** | | **N=286** | | **N=1060** | |
| Less than 200,000 NOK | **7** | 2.5 % | **6** | 2.3 % | **3** | 1.3 % | **7** | 2.5 % | **23** | 2.2 % |
| 200,000 to 399,999 NOK | **21** | 7.6 % | **22** | 8.5 % | **25** | 10.4 % | **24** | 8.4 % | **92** | 8.7 % |
| 400,000 to 599,999 NOK | **40** | 14.5 % | **49** | 19.0 % | **38** | 15.8 % | **42** | 14.7 % | **169** | 15.9 % |
| 600,000 to 799,999 NOK | **61** | 22.1 % | **67** | 26.0 % | **77** | 32.1 % | **55** | 19.2 % | **260** | 24.5 % |
| 800 000 to 1,399,00 NOK | **97** | 35.1 % | **83** | 32.2 % | **66** | 27.5 % | **110** | 38.5 % | **356** | 33.6 % |
| 1,400,000 or more | **11** | 4.0 % | **11** | 4.3 % | **10** | 4.2 % | **3** | 1.1 % | **35** | 3.3 % |
| *(missing / did not answer)* | **39** | 14.1 % | **20** | 7.8 % | **21** | 8.8 % | **45** | 15.7 % | **125** | 11.8 % |
|  |  |  |  |  |  |  |  |  |  |  |
| **Geographic location** | **N=276** | | **N=258** | | **N=240** | | **N=286** | | **N=1060** | |
| Oslo | **64** | 23.2 % | **56** | 21.7 % | **64** | 26.7 % | **76** | 26.6 % | **260** | 24.5 % |
| Southeastern Norway (excl. Oslo) | **84** | 30.4 % | **67** | 26.0 % | **67** | 27.9 % | **71** | 24.8 % | **289** | 27.3 % |
| South West | **77** | 27.9 % | **90** | 34.9 % | **62** | 25.8 % | **82** | 28.7 % | **311** | 29.3 % |
| North | **51** | 18.5 % | **45** | 17.4 % | **47** | 19.6 % | **57** | 19.9 % | **200** | 18.9 % |
|  |  |  |  |  |  |  |  |  |  |  |
| **Marital status** | **N=276** | | **N=258** | | **N=240** | | **N=286** | | **N=1060** | |
| Married/cohabitating | **199** | 72.1 % | **173** | 67.1 % | **152** | 63.3 % | **201** | 70.3 % | **725** | 68.4 % |
| Single | **77** | 27.9 % | **85** | 33.0 % | **87** | 36.3 % | **85** | 29.7 % | **334** | 31.5 % |
| *(missing / did not answer)* | **0** | 0.0 % | **0** | 0.0 % | **1** | 0.4 % | **0** | 0.0 % | **1** | 0.1 % |
|  |  |  |  |  |  |  |  |  |  |  |
| **Education** | **N=276** | | **N=258** | | **N=240** | | **N=286** | | **N=1060** | |
| ≤High school | **118** | 42.8 % | **119** | 46.1 % | **93** | 38.8 % | **133** | 46.5 % | **463** | 43.7 % |
| ≥1 year of university or college | **158** | 57.3 % | **139** | 53.9 % | **147** | 61.3 % | **153** | 53.5 % | **597** | 56.3 % |
|  |  |  |  |  |  |  |  |  |  |  |
| **Born in Norway** | **N=276** | | **N=258** | | **N=240** | | **N=286** | | **N=1060** | |
| Yes | **251** | 90.9 % | **246** | 95.4 % | **228** | 95.0 % | **274** | 95.8 % | **999** | 94.3 % |
| No | **25** | 9.1 % | **12** | 4.7 % | **11** | 4.6 % | **12** | 4.2 % | **60** | 5.7 % |
| *(missing / did not answer)* | **0** | 0.0 % | **0** | 0.0 % | **1** | 0.4 % | **0** | 0.0 % | **1** | 0.1 % |
|  |  |  |  |  |  |  |  |  |  |  |
|  |  |  |  |  |  |  |  |  |  |  |
| **Ever had a Pap test done?** | **N=276** | | **N=258** | | **N=240** | | **N=286** | | **N=1060** | |
| Yes | **262** | 94.9 % | **245** | 95.0 % | **228** | 95.0 % | **275** | 96.2 % | **1010** | 95.3 % |
| No | **11** | 4.0 % | **8** | 3.1 % | **11** | 4.6 % | **9** | 3.2 % | **39** | 3.7 % |
| Don't know | **2** | 0.7 % | **5** | 1.9 % | **1** | 0.4 % | **2** | 0.7 % | **3** | 0.3 % |
| *(missing / did not answer)* | **1** | 0.4 % | **0** | 0.0 % | **0** | 0.0 % | **0** | 0.0 % | **8** | 0.8 % |
|  |  |  |  |  |  |  |  |  |  |  |
| **Hysterectomized** | **N=276** | | **N=258** | | **N=240** | | **N=286** | | **N=1060** | |
| Yes | **23** | 8.3 % | **13** | 5.0 % | **13** | 5.4 % | **18** | 6.3 % | **67** | 6.3 % |
| No | **253** | 91.7 % | **242** | 93.8 % | **226** | 94.2 % | **268** | 93.7 % | **989** | 93.3 % |
| Don't know | **0** | 0.0 % | **1** | 0.4 % | **0** | 0.0 % | **0** | 0.0 % | **1** | 0.1 % |
| *(missing / did not answer)* | **0** | 0.0 % | **2** | 0.8 % | **1** | 0.4 % | **0** | 0.0 % | **3** | 0.3 % |
|  |  |  |  |  |  |  |  |  |  |  |
| **Planning future pregnancy?** | **N=276** | | **N=258** | | **N=240** | | **N=286** | | **N=1060** | |
| Yes | **37** | 13.4 % | **30** | 11.6 % | **21** | 8.8 % | **39** | 13.6 % | **127** | 12.0 % |
| No | **225** | 81.5 % | **216** | 83.7 % | **203** | 84.6 % | **235** | 82.2 % | **879** | 82.9 % |
| Don't know | **14** | 5.1 % | **12** | 4.7 % | **14** | 5.8 % | **12** | 4.2 % | **52** | 4.9 % |
| *(missing / did not answer)* | **0** | 0.0 % | **0** | 0.0 % | **2** | 0.8 % | **0** | 0.0 % | **2** | 0.2 % |

**Table** **A2b**: Baseline characteristics of surveyed Norwegian women adjusted by post-stratification weight.

|  | **Group 1** | | **Group 2** | | **Group 3** | | **Group 4** | |  |  |
| --- | --- | --- | --- | --- | --- | --- | --- | --- | --- | --- |
| **Variables** | No additional info (control) | | Additional info at treatment | | Additional info at abnormal results | | Additional info at initial invitation | | **Total** | |
|  |  | |  | |  | |  | |  | |
|  | **N=273** | | **N=265** | | **N=240** | | **N=282** | | **N=1060** | |
| **Age (years), mean (SD)** | 45.48 | (11,6) | 47.05 | (12,2) | 46.38 | (11,8) | 44.5 | (11,7) | 45.81 | (11,9) |
|  |  |  |  |  |  |  |  |  |  |  |
| **Age groups** | **N=272,7** | | **N=265,2** | | **N=240.2** | | **N=281,8** | | **N=1060** | |
| <30 | 12.6 | 4.6 % | 15.1 | 5.7 % | 14.0 | 5.8 % | 22.3 | 7.9 % | 64.0 | 6.0 % |
| 30-39 | 84.7 | 31.0 % | 71.4 | 26.9 % | 64.8 | 27.0 % | 93.1 | 33.0 % | 314.0 | 29.7 % |
| 40-49 | 76.9 | 28.2 % | 65.7 | 24.8 % | 67.4 | 28.1 % | 65.0 | 23.1 % | 275.0 | 26.0 % |
| 50-59 | 60.4 | 22.1 % | 55.8 | 21.0 % | 51.2 | 21.3 % | 70.0 | 24.9 % | 237.4 | 22.4 % |
| 60-69 | 38.3 | 14.0 % | 57.2 | 21.6 % | 42.9 | 17.8 % | 31.4 | 11.1 % | 169.6 | 16.0 % |
|  |  |  |  |  |  |  |  |  |  |  |
| **Household annual income (NOK)** | **N=232,6** | | **N=245,1** | | **N=218** | | **N=237,5** | | **N=933,1** | |
| *Less than 200,000 NOK* | 7.4 | 3.2 % | 6.3 | 2.6 % | 3.0 | 1.4 % | 7.9 | 17.3 % | 24.6 | 2.6 % |
| *200,000 to 399,999 NOK* | 21.8 | 9.4 % | 24.5 | 10.0 % | 28.2 | 12.9 % | 24.2 | 14.4 % | 98.7 | 10.6 % |
| *400,000 to 599,999 NOK* | 39.6 | 17.0 % | 49.1 | 20.0 % | 36.7 | 16.8 % | 41.3 | 18.3 % | 166.6 | 17.9 % |
| *600,000 to 799,999 NOK* | 58.2 | 25.0 % | 72.2 | 29.4 % | 76.0 | 34.8 % | 54.1 | 17.3 % | 260.5 | 27.9 % |
| *800 000 to 1,399,00 NOK* | 95.7 | 41.1 % | 83.0 | 33.9 % | 64.4 | 29.5 % | 107.1 | 26.1 % | 350.2 | 37.5 % |
| *1,400,000 or more* | 9.9 | 4.3 % | 10.1 | 4.1 % | 9.8 | 4.5 % | 2.9 | 6.7 % | 32.7 | 3.5 % |
|  |  |  |  |  |  |  |  |  |  |  |
| **Geographic location** | **N=272,7** | | **N=265,2** | | **N=240,2** | | **N=281,8** | | **N=1060** | |
| Oslo | 64.7 | 23.7 % | 57.6 | 21.7 % | 64.4 | 26.8 % | 75.8 | 26.9 % | 262.4 | 24.8 % |
| Southeastern Norway (excluding Oslo) | 79.6 | 29.2 % | 67.1 | 25.3 % | 63.4 | 26.4 % | 66.8 | 23.7 % | 277.0 | 26.1 % |
| South West | 79.7 | 29.2 % | 95.0 | 35.8 % | 66.2 | 27.5 % | 86.2 | 30.6 % | 327.1 | 30.9 % |
| North | 48.7 | 17.9 % | 45.5 | 17.2 % | 46.3 | 19.3 % | 53.0 | 18.8 % | 193.5 | 18.3 % |
|  |  |  |  |  |  |  |  |  |  |  |
| **Marital status** | **N=272,7** | | **N=265,2** | | **N=239,3** | | **N=281,8** | | **N=1059** | |
| Married/cohabitating | 197.7 | 72.5 % | 178.2 | 67.2 % | 152.2 | 63.6 % | 196.0 | 69.6 % | 724.2 | 68.4 % |
| Single | 75.1 | 27.5 % | 87.0 | 32.8 % | 87.0 | 36.4 % | 85.8 | 30.5 % | 334.9 | 31.6 % |
|  |  |  |  |  |  |  |  |  |  |  |
| **Education** | **N=272,7** | | **N=265,2** | | **N=240,2** | | **N=281,8** | | **N=1060** | |
| ≤High school | 119.1 | 43.7 % | 120.2 | 45.3 % | 91.0 | 37.9 % | 126.7 | 45.0 % | 456.9 | 43.1 % |
| ≥1 year of university or college | 153.6 | 56.3 % | 145.1 | 54.7 % | 149.3 | 62.1 % | 155.1 | 55.0 % | 603.1 | 56.9 % |
|  |  |  |  |  |  |  |  |  |  |  |
| **Born in Norway** | **N=272,7** | | **N=265,2** | | **N=239,3** | | **N=281,8** | | **N=1059** | |
| Yes | 247.4 | 90.7 % | 255.1 | 96.2 % | 229.1 | 95.7 % | 269.8 | 95.8 % | 1001.0 | 94.6 % |
| No | 25.4 | 9.3 % | 10.2 | 3.8 % | 10.2 | 4.3 % | 12.0 | 4.3 % | 57.7 | 5.5 % |
|  |  |  |  |  |  |  |  |  |  |  |
| **Ever had a Pap test done?** | **N=272** | | **N=260** | | **N=240,2** | | **N=280** | | **N=1052** | |
| Yes | 257.0 | 94.5 % | 250.6 | 96.4 % | 226.8 | 94.4 % | 269.4 | 96.2 % | 1004.0 | 95.4 % |
| No | 12.9 | 4.8 % | 9.5 | 3.7 % | 12.4 | 5.2 % | 10.6 | 3.8 % | 45.4 | 4.3 % |
| Don't know | 2.1 | 0.8 % | 0.0 | 0.0 % | 1.1 | 0.5 % | 0.0 | 0.0 % | 3.2 | 0.3 % |
|  |  |  |  |  |  |  |  |  |  |  |
| **Hysterectomized** | **N=272,7** | | **N=263,7** | | **N=239,5** | | **N=281,8** | | **N=1058** | |
| Yes | 20.3 | 7.5 % | 13.6 | 5.1 % | 12.5 | 5.2 % | 16.8 | 6.0 % | 63.2 | 6.0 % |
| No | 252.4 | 92.6 % | 249.1 | 94.4 % | 227.0 | 94.8 % | 265.0 | 94.0 % | 993.5 | 93.9 % |
| Don't know | 0.0 | 0.0 % | 1.1 | 0.4 % | 0.0 | 0.0 % | 0.0 | 0.0 % | 1.1 | 0.1 % |
|  |  |  |  |  |  |  |  |  |  |  |
| **Planning future pregnancy?** | **N=272,7** | | **N=265,2** | | **N=238,6** | | **N=281,8** | | **N=1058** | |
| Yes | 42.4 | 15.6 % | 35.8 | 13.5 % | 24.5 | 10.3 % | 45.8 | 16.2 % | 148.4 | 14.0 % |
| No | 215.2 | 78.9 % | 215.4 | 81.2 % | 198.7 | 83.3 % | 221.6 | 78.6 % | 850.8 | 80.4 % |
| Don't know | 15.2 | 5.6 % | 14.0 | 5.3 % | 15.4 | 6.5 % | 14.5 | 5.1 % | 59.1 | 5.6 % |

**Table A2c**: Baseline characteristics of surveyed Norwegian women adjusted by post-stratification weight compared to the Norwegian population distribution

|  |  |  |  |  |  |
| --- | --- | --- | --- | --- | --- |
|  | **Norway Survey Population** | | | **Norway Population*** | |
|  |  |  |  |  |  |
|  | **Age groups** | **N=1060** | |  | **Age groups** |
|  | <30 | 64.0 | 6.0 % | 11.1% | <30 |
|  | 30-39 | 314.0 | 29.7 % | 23.7% | 30-39 |
|  | 40-49 | 275.0 | 26.0 % | 24.8% | 40-49 |
|  | 50-59 | 237.4 | 22.4 % | 21.8% | 50-59 |
|  | 60-69 | 169.6 | 16.0 % | 18.6% | 60-69 |
|  |  |  |  |  |  |
|  | **Personal annual income (NOK)^a^** | **N=919^b^** | |  | **Personal annual income (NOK)^a^** |
|  | Less than 200,000 NOK | 86.0 | 9.4 % | 22.5 % | Less than 200,000 NOK |
|  | 200,000 to 399,999 NOK | 428.3 | 46.6 % | 48.0 % | 200,000 to 399,999 NOK |
|  | 400,000 to 599,999 NOK | 338.1 | 36.8 % | 20.8 % | 400,000 to 599,999 NOK |
|  | 600,000 to 799,999 NOK | 54.7 | 6.0 % | 6.2 % | 600,000 to 799,999 NOK |
|  | Over 800 000 NOK | 11.9 | 1.3 % | 2.5 % | Over 800 000 NOK |
|  |  |  |  |  |  |
|  |  |  |  |  |  |
|  | **Geographic location** | **N=1060** | |  | **Geographic location** |
|  | Oslo | 262.4 | 24.8 % | 27.8 % | Oslo |
|  | Southeastern Norway (excluding Oslo) | 277.0 | 26.1 % | 25.1 % | Southeastern Norway (excluding Oslo) |
|  | South West | 327.1 | 30.9 % | 30.9 % | South West |
|  | North | 193.5 | 18.3 % | 16.2 % | North |
|  |  |  |  |  |  |
|  | **Marital status** | **N=1059** | |  | **Marital status** |
|  | Married/cohabitating | 724.2 | 68.4 % | 71.3% | Married/cohabitating |
|  | Single | 334.9 | 31.6 % | 28.7 % | Single |
|  |  |  |  |  |  |
|  | **Education** | **N=1060** | |  | **Education** |
|  | ≤High school | 456.9 | 43.1 % | 61.0 % | ≤High school |
|  | ≥1 year of university or college | 603.1 | 56.9 % | 39.0 % | ≥1 year of university or college |
|  |  |  |  |  |  |
|  | **Born in Norway** | **N=1059** | |  | **Born in Norway** |
|  | Yes | 1001.0 | 94.6 % | 89.0 % | Yes |
|  | No | 57.7 | 5.5 % | 11.0 % | No |
|  |  |  |  |  |  |
|  |  |  |  |  |  |
|  |  |  |  |  |  |
| * | Data from Statistics Norway (SSB) | | | | |
| ^a^ | Household income tables 2013 no longer available from SSB, we instead used personal income (previously reported in Iyer et al, 2019) | | | | |
| ^b^ | Lower (n) due to women choosing "prefer not to answer" | | |  |  |

**Table A3:** Baseline knowledge of CC for both US and Norwegian Women. The first question asked women to identify the three most common cancers in their country and was regarded as wrong if women answered CC as one of their choice and as correct if they did not select CC. The second question was also asked as a multiple choice with 5 alternative or “don’t know” with only one answer being correct and all other answers, including those who answered “don’t know” were coded as wrong. Frequencies of answers have been adjusted with post-stratification weights.

|  | **Which three do you think are the three most common types of cancer among women in the USA?** | | | |
| --- | --- | --- | --- | --- |
|  |  |  |  |  |
|  |  |  | American Women (n=1084) | Norwegian Women (n=1059) |
|  | Correct |  | 37.5 % | 11.1 % |
|  | (Wrong) CC |  | 62.5 % | 88.9 % |
|  |  |  |  |  |
|  | **What do you think is the main reason that some women develop cervical cancer?** | | | |
|  |  |  |  |  |
|  | Family history |  | 29.6 % | 21.5 % |
|  | (correct) Virus |  | 33.2 % | 42.2 % |
|  | Hormones |  | 5.3 % | 13.2 % |
|  | Smoking |  | 1.3 % | 0.4 % |
|  | Other |  | 3.4 % | 2.2 % |
|  | Don't know |  | 27.2 % | 20.5 % |
|  |  |  |  |  |
|  | Virus (correct) |  | 33.2 % | 42.2 % |
|  | Pooled (incorrect) |  | 66.8 % | 57.8 % |

**Table A4.** Primary results of Norwegian women's intention to screen, follow-up abnormal results and follow-up with precancer treatment by information group. Values are percentages (95% confidence intervals) unless otherwise stated.

|  | | | | | | |
| --- | --- | --- | --- | --- | --- | --- |
|  |  | **Information Groups** | | | |  |
|  | **Variables*** | **No additional information**  **(% and 95% CI).** | | **Additional information**  **(% and 95% CI).** | | **p-value** |
|  |  |  |  |  |  |  |
|  | Intend to participate in CC screening? | (N=725) | | (N=268) | |  |
|  | Yes | 88.0 | (85.5 - 90.2) | 87.1 | (82.2 - 90.8) |  |
|  | No | 3.7 | (2.5 - 5.5) | 2.2 | (1.0 - 4.9) |  |
|  | Don't know | 8.3 | (6.5 - 10.7) | 10.7 | (7.3 - 15.4) | 0.298^a^ |
|  | How likely to participate? (scale 1-10)^X^ |  |  |  |  |  |
|  | Mean (CI) | 8.9 | (8.8 - 9.1) | 8.7 | (8.4 - 8.9) | 0.281^Y^ |
|  | Intend to participate in follow-up control? | (N=496) | | (N=495) | |  |
|  | Yes | 96.9 | (94.8 - 98.2) | 96.4 | (94.3 - 97.8) |  |
|  | No | 0.0 | (0 - 0) | 0.1 | (0.002 - 2.1) |  |
|  | Don't know | 3.1 | (1.8 - 5.2) | 2.8 | (1.6 - 4.8) | 0.150^b^ |
|  | How likely to participate in follow-up? (scale 1-10)^X^ |  |  |  |  |  |
|  | Mean (CI) | 9.5 | (9.4 - 9.7) | 9.5 | (9.4 - 9.6) | 0.260^Y^ |
|  | Intention to follow-up with surgical treatment | (N=253) | | (N=740) | |  |
|  | Yes | 92.1 | (87.7 - 95.1) | 84.6 | (81.7 - 87.2) |  |
|  | No | 0.0 | (0 - 0) | 1.0 | (0.5 - 2.2) |  |
|  | Don't know | 7.9 | (5.0 - 12.3) | 14.3 | (11.9 - 17.2) | 0.012^c^ |
|  | How likely to follow-up with precancer treatment? (scale 1-10)^X^ |  |  |  |  |  |
|  | Mean (CI) | 9.4 | (9.2 - 9.6) | 9.0 | (8.9 - 9.1) | 0.003^Y^ |
| CC. cervical cancer; CI. confidence interval | | | | | | |
| **^*^** A maximum of 2 women (0.2%) chose not to answer on any given question. | | | | | | |
| ^X^ CI for nonparametric distribution calculated by bootstrapping and using bias-corrected estimates | | | | | | |
| ^Y^ Kruskal-Wallis test on unweighted data | | | | | | |
| ^a Yes vs. No: P-value=0.268; Yes vs. Don't Know: P-value=0.302; No vs. Don't know: P-value=0.135^ | | | | | | |
| ^b Yes vs. No: P-value=0.048; Yes vs. Don't Know: P-value=0.804; No vs. Don't know: P-value=0.058^ | | | | | | |
| ^c Yes vs. No: P-value=0.112; Yes vs. Don't Know: P-value=0.012; No vs. Don't know: P-value=0.203^ | | | | | | |

**Table A5.** Primary results of US women's intention to screen, follow-up abnormal results, and follow-up with precancer treatment by randomized group. Values are percentages (95% confidence intervals) unless otherwise stated.

|  | | | | | | |
| --- | --- | --- | --- | --- | --- | --- |
|  |  | **Information Groups** | | | |  |
|  | **Variables*** | **No additional information**  **(% and 95% CI)** | | **Additional information**  **(% and 95% CI)** | | **p-value** |
|  |  |  |  |  |  |  |
|  | Intend to participate in CC screening? | (N=683) | | (N=234) | |  |
|  | Yes | 76.4 | (72.3 - 80.0) | 70.2 | (62.0 - 77.2) |  |
|  | No | 6.0 | (4.2 - 8.3) | 8.3 | (4.6 - 14.5) |  |
|  | Don't know | 17.7 | (14.4 - 21.5) | 24.6 | (15.5 - 29.2) | 0.321^a^ |
|  | How likely to participate? (scale 1-10)^X^ |  |  |  |  |  |
|  | Mean (CI) | 8.2 | (8.1 - 8.4) | 8.2 | (7.6 - 8.3) | 0.195^Y^ |
|  | Intend to participate in follow-up control? | (N=458) | | (N=459) | |  |
|  | Yes | 92.0 | (87.8 - 94.9) | 89.9 | (85.3 - 93.1) |  |
|  | No | 2.1 | (0.9 - 4.8) | 0.7 | (0.3 - 1.6) |  |
|  | Don't know | 5.8 | (3.4 - 9.7) | 9.5 | (6.2 -14.1) | 0.089^b^ |
|  | How likely to participate in follow-up? (scale 1-10)^X^ |  |  |  |  |  |
|  | Mean (CI) | 9.2 | (9.0 - 9.3) | 8.9 | (8.7 - 9.2) | 0.316^Y^ |
|  | Intention to follow-up with surgical treatment | (N=238) | | (N=679) | |  |
|  | Yes | 70.7 | (62.6 - 77.7) | 69.1 | (64.6 - 73.4) |  |
|  | No | 3.8 | (1.6 - 8.6) | 5.6 | (3.7 - 8.2) |  |
|  | Don't know | 25.6 | (19.0 - 33.5) | 25.3 | (21.4 - 29.7) | 0.707^c^ |
|  | How likely to follow-up with precancer treatment? (scale 1-10)^X^ |  |  |  |  |  |
|  | Mean (CI) | 8.9 | (8.6 - 9.1) | 8.4 | (8.6 - 8.5) | 0.001^Y^ |
| CC. cervical cancer | | | | | | |
| **^*^** A maximum of 3 women (0.3%) chose not to answer on any given question. | | | | | | |
| ^X^ CI for nonparametric distribution calculated by bootstrapping and using bias-corrected estimates | | | | | | |
| ^Y^ Kruskal-Wallis test on unweighted data | | | | | | |
| ^a Yes vs. No: P-value=0.264; Yes vs. Don't Know: P-value=0.243; No vs. Don't know: P-value=0.754^ | | | | | | |
| ^b Yes vs. No: P-value=0.055; Yes vs. Don't Know: P-value=0.168; No vs. Don't know: P-value=0.016^ | | | | | | |
| ^c Yes vs. No: P-value=0.406; Yes vs. Don't Know: P-value=0.960; No vs. Don't know: P-value=0.445^ | | | | | | |

**Table A6**

Multivariable logistic regressions for the three questions on intention to participate and follow recommendations at each of the three simplified steps of the screening process. The dependent variable, intention to participate, is coded as 1=yes, 0=no or don’t know. Odds ratios are shownas well as the average marginal effect (AME). Women that had a hysterectomy were excluded from the analysis. The income categories are listed in US dollars and were adjusted for purchasing power parity. The ethnicity of Norwegian women was not available in the dataset, therefore the variable “born in Norway” was used as a proxy. All analyses were conducted on the data adjusted with post-stratification weights. **p-value <0.01, *p-value <0.05.

**Table A6 (IN TEXT FORMAT / BROKEN INTO 3 PANELS)**

|  | **1 - Participation in routine screening** | | | | | | | | | | | | |
| --- | --- | --- | --- | --- | --- | --- | --- | --- | --- | --- | --- | --- | --- |
|  |  |  |  |  |  |  |  |  |  |  |  |  |  |
|  | **Norwegian Women** | | | | | |  | **American Women** | | | | | |
|  |  |  |  |  |  |  |  |  |  |  |  |  |  |
| ***Binomial Logistic Regression*** | ***Univariate*** | | ***Multivariable*** | | ***Average*** | |  | ***Univariate*** | | ***Multivariable*** | | ***Average*** | |
|  | *odds ratio* | | *odds ratio* | | ***Margin. Effect*** | |  | *odds ratio* | | *odds ratio* | | ***Margin. Effect*** | |
| **Additional information received** |  |  |  |  |  |  |  |  |  |  |  |  |  |
| *Additional information received* | 0.923 |  | 1.013 |  | 0.11 | * |  | 0.727 |  | 0.766 |  | -4.58 |  |
| *No add. information (control)* | *ref.* |  | *ref.* |  | *ref.* |  |  | *ref.* |  | *ref.* |  | *ref.* |  |
| **Income** |  |  |  |  |  |  |  |  |  |  |  |  |  |
| *Less than $24,999* | 2.169 |  | 3.451 |  | 11.48 |  |  | 0.454 |  | 0.739 |  | -4.64 |  |
| *$25,000 to $39,999* | 0.737 |  | 0.996 |  | -0.05 |  |  | 0.353 | * | 0.510 |  | -11.28 |  |
| *$40,000 to $59,999* | 1.680 |  | 2.359 |  | 8.98 |  |  | 0.491 |  | 0.551 |  | -9.82 |  |
| *$60,000 to $84,999* | 1.340 |  | 1.963 |  | 7.48 |  |  | 0.589 |  | 0.627 |  | -7.46 |  |
| *$85,000 to $149,999* | 2.220 |  | 2.515 |  | 9.45 |  |  | 0.859 |  | 0.913 |  | -1.33 |  |
| *$150,000 or more* | *ref.* |  | *ref.* |  | *ref.* |  |  | *ref.* |  | *ref.* |  | *ref.* |  |
| **Age** |  |  |  |  |  |  |  |  |  |  |  |  |  |
| *age 20-29* | 1.249 |  | 7.044 | ** | 13.55 | ** |  | 0.809 |  | 0.865 |  | -2.54 |  |
| *age 30-39* | 1.560 |  | 2.551 | ** | 8.82 |  |  | 1.592 |  | 1.266 |  | 3.83 |  |
| *age 40-49* | 1.725 |  | 1.524 |  | 4.65 |  |  | 1.125 |  | 1.029 |  | 0.48 |  |
| *age 50-59* | 1.838 |  | 1.660 |  | 5.45 |  |  | 1.068 |  | 1.042 |  | 0.70 |  |
| *age 60+* | *ref.* |  | *ref.* |  | *ref.* |  |  | *ref.* |  | *ref.* |  | *ref.* |  |
| **Marital status** |  |  |  |  |  |  |  |  |  |  |  |  |  |
| *Single* | 0.677 |  | 0.998 |  | -0.02 |  |  | 0.483 | ** | 0.635 | * | -7.94 | * |
| *Married or Cohabitating* | *ref.* |  | *ref.* |  | *ref.* |  |  | *ref.* |  | *ref.* |  | *ref.* |  |
| **Education** |  |  |  |  |  |  |  |  |  |  |  |  |  |
| *No university or college* | 0.679 |  | 0.945 |  | -0.49 |  |  | 0.539 | ** | 0.675 |  | -6.79 |  |
| *University or college* | *ref.* |  | *ref.* |  | *ref.* |  |  | *ref.* |  | *ref.* |  | *ref.* |  |
| **Cause of CC is a virus** |  |  |  |  |  |  |  |  |  |  |  |  |  |
| *HPV* | 1.12 |  | 0.837 |  | -1.53 |  |  | 1.7556 | ** | 1.427 |  | 5.85 |  |
| *incorrect* | *ref.* |  | *ref.* |  | *ref.* |  |  | *ref.* |  | *ref.* |  | *ref.* |  |
| **Ethnicity** |  |  |  |  |  |  |  |  |  |  |  |  |  |
| *African-American/Black* | *-* |  | *-* |  | *-* |  |  | 0.993 |  | 1.238 |  | 3.67 |  |
| *Hispanic or Latino* | *-* |  | *-* |  | *-* |  |  | 1.187 |  | 1.776 |  | 9.13 |  |
| *Other* | *-* |  | *-* |  | *-* |  |  | 1.916 |  | 2.310 | * | 12.50 | * |
| Caucasian | *-* |  | *-* |  | *-* |  |  | *ref* |  | *ref* |  | *ref* |  |
| **Born in Norway** |  |  |  |  |  |  |  |  |  |  |  |  |  |
| *Born in Norway (Yes)* | 1.391 |  | 0.754 |  | -2.20 |  |  | *-* |  | *-* |  | *-* |  |
| (No) | *ref* |  | *ref* |  | *ref.* |  |  | *-* |  | *-* |  | *-* |  |
| **Planning future pregnancy** |  |  |  |  |  |  |  |  |  |  |  |  |  |
| *(No or don't know)* | 1.372 |  | 2.198 |  | 8.36 |  |  | 0.550 | * | 0.585 |  | -8.37 |  |
| (Yes) | *ref* |  | *ref* |  | *ref.* |  |  | *ref* |  | *ref* |  | *ref* |  |
| **Had a previous test before** |  |  |  |  |  |  |  |  |  |  |  |  |  |
| *(No or don't know)* | 0.130 | ** | 0.100 | ** | -37.97 | ** |  | 0.178 | ** | 0.205 | ** | -0.33 | ** |
| (Yes) | *ref* |  | *ref* |  | *ref.* |  |  | *ref* |  | *ref* |  | *ref* |  |

|  | **2 - Follow-up on abnormal results (control Pap-smear)** | | | | | | | | | | | | |
| --- | --- | --- | --- | --- | --- | --- | --- | --- | --- | --- | --- | --- | --- |
|  |  |  |  |  |  |  |  |  |  |  |  |  |  |
|  | **Norwegian Women** | | | | | |  | **American Women** | | | | | |
|  |  |  |  |  |  |  |  |  |  |  |  |  |  |
| ***Binomial Logistic Regression*** | ***Univariate*** | | ***Multivariable*** | | ***Average*** | |  | ***Univariate*** | | ***Multivariable*** | | ***Average*** | |
|  | *odds ratio* | | *odds ratio* | | ***Margin. Effect*** | |  | *odds ratio* | | *odds ratio* | | ***Margin. Effect*** | |
| **Additional information received** |  |  |  |  |  |  |  |  |  |  |  |  |  |
| *Additional information received* | 0.856 |  | 0.841 |  | -0.50 |  |  | 0.768 |  | 0.834 |  | -1.21 |  |
| *No add. information (control)* | *ref.* |  | *ref.* |  | *ref* |  |  | *ref.* |  | *ref.* |  | *ref* |  |
| **Income** |  |  |  |  |  |  |  |  |  |  |  |  |  |
| *Less than $24,999* | 0.336 |  | 0.649 |  | -1.73 |  |  | 0.087 | ** | 0.085 | ** | -12.53 | ** |
| *$25,000 to $39,999* | 0.268 |  | 0.452 |  | -3.73 |  |  | 0.152 | ** | 0.141 | * | -8.07 | * |
| *$40,000 to $59,999* | 0.746 |  | 0.996 |  | 2.17 |  |  | 0.118 | ** | 0.091 | ** | -11.89 | ** |
| *$60,000 to $84,999* | 1.681 |  | 2.662 |  | 0.92 |  |  | 0.279 |  | 0.198 |  | -5.76 | * |
| *$85,000 to $149,999* | 1.221 |  | 1.377 |  |  |  |  | 0.400 |  | 0.337 |  |  |  |
| *$150,000 or more* | *ref.* |  | *ref.* |  | *ref* |  |  | *ref.* |  | *ref.* |  | *ref* |  |
| **Age** |  |  |  |  |  |  |  |  |  |  |  |  |  |
| *age 20-29* | 1.213 |  | 2.531 |  | 2.22 |  |  | 0.507 |  | 0.605 |  | -4.65 |  |
| *age 30-39* | 0.958 |  | 0.930 |  | -0.26 |  |  | 1.351 |  | 1.620 |  | 3.32 |  |
| *age 40-49* | 2.142 |  | 1.414 |  | 1.04 |  |  | 1.087 |  | 1.142 |  | 1.02 |  |
| *age 50-59* | 1.748 |  | 1.601 |  | 1.34 |  |  | 3.283 | * | 4.549 | ** | 7.56 | * |
| *age 60+* | *ref.* |  | *ref.* |  | *ref* |  |  | *ref.* |  | *ref.* |  | *ref* |  |
| **Marital status** |  |  |  |  |  |  |  |  |  |  |  |  |  |
| *Single* | 0.633 |  | 0.928 |  | -0.22 |  |  | 0.912 |  | 2.451 | * | 5.58 | ** |
| *Married or Cohabitating* | *ref.* |  | *ref.* |  | *ref* |  |  | *ref.* |  | *ref.* |  | *ref* |  |
| **Education** |  |  |  |  |  |  |  |  |  |  |  |  |  |
| *No university or college* | 0.732 |  | 0.559 |  | -1.72 |  |  | 0.436 | ** | 0.534 |  | -4.33 |  |
| *University or college* | *ref.* |  | *ref.* |  | *ref* |  |  | *ref.* |  | *ref.* |  | *ref* |  |
| **Cause of CC is a virus** |  |  |  |  |  |  |  |  |  |  |  |  |  |
| *HPV* | 1.136 |  | 0.9576 |  | -0.13 |  |  | 3.276 | ** | 1.823 |  | 3.72 |  |
| *incorrect* | *ref.* |  | *ref.* |  | *ref* |  |  | *ref.* |  | *ref.* |  | *ref* |  |
| **Ethnicity** |  |  |  |  |  |  |  |  |  |  |  |  |  |
| *African-American/Black* | *-* |  | *-* |  | *-* |  |  | 0.364 | * | 0.427 |  | -6.80 |  |
| *Hispanic or Latino* | *-* |  | *-* |  | *-* |  |  | 0.989 |  | 2.106 |  | 3.59 |  |
| *Other* | *-* |  | *-* |  | *-* |  |  | 0.312 | * | 0.273 | * | -11.82 |  |
| Caucasian | *-* |  | *-* |  | *-* |  |  | *ref* |  | *ref* |  | *ref* |  |
| **Born in Norway** |  |  |  |  |  |  |  |  |  |  |  |  |  |
| *Born in Norway (Yes)* | 2.473 |  | 2.602 |  | 3.98 |  |  | *-* |  | *-* |  | *-* |  |
| (No) | *ref* |  | *ref* |  | *ref* |  |  | *-* |  | *-* |  | *-* |  |
| **Planning future pregnancy** |  |  |  |  |  |  |  |  |  |  |  |  |  |
| *(No or don't know)* | 1.366 |  | 0.984 |  | -0.05 |  |  | 0.946 |  | 0.647 |  | -2.70 |  |
| (Yes) | *ref* |  | *ref* |  | *ref* |  |  | *ref* |  | *ref* |  | *ref* |  |
| **Had a previous test before** |  |  |  |  |  |  |  |  |  |  |  |  |  |
| *(No or don't know)* | 0.184 | ** | 0.118 | ** | -14.18 |  |  | 0.141 | ** | 0.160 | ** | -19.36 | * |
| (Yes) | *ref* |  | *ref* |  | *ref* |  |  | *ref* |  | *ref* |  | *ref* |  |

|  | **3 - Follow-up with surgical treatment** | | | | | | | | | | | | |
| --- | --- | --- | --- | --- | --- | --- | --- | --- | --- | --- | --- | --- | --- |
|  |  |  |  |  |  |  |  |  |  |  |  |  |  |
|  | **Norwegian Women** | | | | | |  | **American Women** | | | | |  |
|  |  |  |  |  |  |  |  |  |  |  |  |  |  |
| ***Binomial Logistic Regression*** | ***Univariate*** | | ***Multivariable*** | | ***Average*** | |  | ***Univariate*** | | ***Multivariable*** | | ***Average*** | |
|  | *odds ratio* | | *odds ratio* | | ***Margin. Effect*** | |  | *odds ratio* | | *odds ratio* | | ***Margin. Effect*** | |
| **Additional information received** |  |  |  |  |  |  |  |  |  |  |  |  |  |
| *Additional information received* | 0.4705 | ** | 0.373 | ** | -8.26 | ** |  | 0.930 |  | 0.955 |  | -0.87 |  |
| *No add. information (control)* | *ref.* |  | *ref.* |  | *ref* |  |  | *ref.* |  | *ref.* |  | *ref* |  |
| **Income** |  |  |  |  |  |  |  |  |  |  |  |  |  |
| *Less than $24,999* | 0.288 |  | 0.499 |  | -6.46 |  |  | 0.295 | ** | 0.480 |  | -12.80 |  |
| *$25,000 to $39,999* | 0.294 |  | 0.455 |  | -7.57 |  |  | 0.252 | ** | 0.379 | * | -17.73 | * |
| *$40,000 to $59,999* | 0.338 |  | 0.459 |  | -7.46 |  |  | 0.407 | * | 0.443 |  | -14.44 | * |
| *$60,000 to $84,999* | 0.400 |  | 0.585 |  | -4.68 |  |  | 0.434 | * | 0.462 |  | -13.61 |  |
| *$85,000 to $149,999* | 0.598 |  | 0.797 |  |  |  |  | 0.634 |  | 0.657 |  |  |  |
| *$150,000 or more* | *ref.* |  | *ref.* |  | *ref* |  |  | *ref.* |  | *ref.* |  | *ref* |  |
| **Age** |  |  |  |  |  |  |  |  |  |  |  |  |  |
| *age 20-29* | 0.734 |  | 1.961 |  | 7.28 |  |  | 0.457 | * | 0.495 |  | -13.25 |  |
| *age 30-39* | 1.179 |  | 1.208 |  | 2.39 |  |  | 0.636 |  | 0.518 | * | -12.29 | * |
| *age 40-49* | 2.238 | * | 1.811 |  | 6.59 |  |  | 1.003 |  | 0.883 |  | -2.08 |  |
| *age 50-59* | 2.323 | * | 2.197 |  | 8.19 |  |  | 0.842 |  | 0.724 |  | -5.65 |  |
| *age 60+* | *ref.* |  | *ref.* |  | *ref* |  |  | *ref.* |  | *ref.* |  | *ref* |  |
| **Marital status** |  |  |  |  |  |  |  |  |  |  |  |  |  |
| *Single* | 0.730 |  | 1.046 |  | 0.46 |  |  | 0.680 | * | 0.902 |  | -1.98 |  |
| *Married or Cohabitating* | *ref.* |  | *ref.* |  | *ref* |  |  | *ref.* |  | *ref.* |  | *ref* |  |
| **Education** |  |  |  |  |  |  |  |  |  |  |  |  |  |
| *No university or college* | 0.739 |  | 0.773 |  | -2.67 |  |  | 0.531 | ** | 0.740 |  | -5.90 |  |
| *University or college* | *ref.* |  | *ref.* |  | *ref* |  |  | *ref.* |  | *ref.* |  | *ref* |  |
| **Cause of CC is a virus** |  |  |  |  |  |  |  |  |  |  |  |  |  |
| *HPV* | 1.135 |  | 1.064 |  | 0.63 |  |  | 2.161 | ** | 1.872 | ** | 11.75 | ** |
| *incorrect* | *ref.* |  | *ref.* |  | *ref* |  |  | *ref.* |  | *ref.* |  | *ref* |  |
| **Ethnicity** |  |  |  |  |  |  |  |  |  |  |  |  |  |
| *African-American/Black* | *-* |  | *-* |  | *-* |  |  | 0.516 | * | 0.676 |  | -7.87 |  |
| *Hispanic or Latino* | *-* |  | *-* |  | *-* |  |  | 0.496 | * | 0.672 |  | -8.02 |  |
| *Other* | *-* |  | *-* |  | *-* |  |  | 1.254 |  | 1.431 |  | 6.29 |  |
| Caucasian | *-* |  | *-* |  | *ref* |  |  | *ref* |  | *ref* |  | *ref* |  |
| **Born in Norway** |  |  |  |  |  |  |  |  |  |  |  |  |  |
| *Born in Norway (Yes)* | 0.756 |  | 1.111 |  | - |  |  | *-* |  | *-* |  | *-* |  |
| (No) | *ref* |  | *ref* |  | *ref* |  |  | *-* |  | *-* |  | *-* |  |
| **Planning future pregnancy** |  |  |  |  |  |  |  |  |  |  |  |  |  |
| *(No or don't know)* | 1.660 | * | 1.682 |  | 6.03 |  |  | 1.129 |  | 0.986 |  | -0.26 |  |
| (Yes) | *ref* |  | *ref* |  | *ref* |  |  | *ref* |  | *ref* |  | *ref* |  |
| **Had a previous test before** |  |  |  |  |  |  |  |  |  |  |  |  |  |
| *(No or don't know)* | 0.292 | ** | 0.426 | * | -11.30 |  |  | 0.309 | ** | 0.452 | * | -16.88 |  |
| (Yes) | *ref* |  | *ref* |  | *ref* |  |  | *ref* |  | *ref* |  | *ref* |  |

**The Norwegian letters:**

The following 6 information letters were used in the study and have been translated from Norwegian.

| **Letter 1, version A**  Recommendation to have a PAP test taken once every three years. | **Letter 1, version B**  Recommendation to have a PAP test taken once every three years. |
| --- | --- |
| **A Pap test can save your life.**  Norwegian health authorities recommend women between 25 and 69 years of age to have a Pap test taken once every three years. The Pap test can detect abnormal cells that may progress to cervical cancer. Effective treatment for severe cell changes is available. The Cancer Registry sends letters to women between 25 and 69 years of age with no Pap tests taken in the past three years and also to women without recommended follow-up of cell changes discovered by screening. More than half of cervical cancers are diagnosed among the minority of women who have not had their Pap test taken. The Cancer Registry registers all Pap test results from the cervix under the provisions of the Cancer  Registry Regulations.  According to our registry, it is more than three years since you last had a Pap test.  We recommend you to make an appointment with your general physician and have a Pap test taken. | **A Pap test can save your life.**  Norwegian health authorities recommend women between 25 and 69 years of age to have a Pap test taken once every three years. The Pap test can detect abnormal cells that may progress to cervical cancer. Effective treatment for severe cell changes is available. The Cancer Registry sends letters to women between 25 and 69 years of age with no Pap tests taken in the past three years and also to women without recommended follow-up of cell changes discovered by screening. More than half of cervical cancers are diagnosed among the minority of women who have not had their Pap test taken. The Cancer Registry registers all Pap test results from the cervix under the provisions of the Cancer  Registry Regulations.  According to our registry, it is more than three years since you last had a Pap test.  We recommend you to make an appointment with your general physician and have a Pap test taken.  ***Benefits and disadvantages to participating in the cervical cancer screening:***  ***Why is it important for me to have a Pap test?*** *Most cell changes in the cervix are caused by Human Papillomaviruses (HPV). HPV infection is very common. HPV occurs both in men and women, and is spread through sexual contact. Most of the HPV infections are harmless and disappear by themselves, but sometimes the infection persists and leads to serious cell changes that can develop into cervical cancer. Treatment is available that effectively reduces the risk of developing cervical cancer. Regular Pap testing (screening) can thereby prevent a large proportion of cancers.*  ***What are the disadvantages of having a Pap test?*** *To participate in a screening program can cause excitement and anxiety until the result of the test is ready. Ask your physician about the advantages and disadvantages of screening or see the web site of The Cancer Registry.*  ***Side effects:*** *Every year, serious cell-changes are detected in 3000 Norwegian women. 2500 of these will make a full recovery without treatment, but we do not know which ones. If the cell-changes are not treated, they can develop into cervical cancer. 1 out of 12 women who have a pap smear taken, has to take one or more control samples due to abnormal cells being found. The cell-changes can be removed with a minor surgical procedure, but this treatment may increase the risk for late-term miscarriage and premature birth in future pregnancies. The surgery is usually recommended to women with two or more abnormal samples.* |

| **Letter 2, version A**  Letter with recommendation for control of cervical pap-smear | **Letter 2, version B**  Letter with recommendation for control of cervical pap-smear |
| --- | --- |
| **- you should take a new pap-smear**  A cervical pap-smear was taken *approximately 6 months ago*. The result of this pap-smear was sent to your doctor along with a recommendation to take a control pap-smear.  We are contacting you now because we have not registered that a control cervical pap-smear has been taken.  A control pap-smear is recommended when the first test showed abnormal cells, or an infection of human papilloma virus (HPV) in the cervix. Such cell-changes usually recede on their own, and most HPV-infections disappear within 6-12 months. We still recommend that you contact your GP to take a control pap-smear.  If you have already taken a control pap-smear, please disregard this letter.  Further information about the Cervical Cancer Screening Programme, including frequently asked questions, can be found at www.kreftregisteret.no/livmorhals | **- you should take a new pap-smear**  A cervical pap-smear was taken *approximately 6 months ago*. The result of this pap-smear was sent to your doctor along with a recommendation to take a control pap-smear.  We are contacting you now because we have not registered that a control cervical pap-smear has been taken.  A control pap-smear is recommended when the first test showed abnormal cells, or an infection of human papilloma virus (HPV) in the cervix. Such cell-changes usually recede on their own, and most HPV-infections disappear within 6-12 months. We still recommend that you contact your GP to take a control pap-smear.  If you have already taken a control pap-smear, please disregard this letter.  ***Benefits and disadvantages to participating in the cervical cancer screening:***  ***Why is it important for me to have a Pap test?*** *Most cell changes in the cervix are caused by Human Papillomaviruses (HPV). HPV infection is very common. HPV occurs both in men and women, and is spread through sexual contact. Most of the HPV infections are harmless and disappear by themselves, but sometimes the infection persists and leads to serious cell changes that can develop into cervical cancer. Treatment is available that effectively reduces the risk of developing cervical cancer. Regular Pap testing (screening) can thereby prevent a large proportion of cancers.*  ***What are the disadvantages of having a Pap test?*** *To participate in a screening program can cause excitement and anxiety until the result of the test is ready. Ask your physician about the advantages and disadvantages of screening or see the web site of The Cancer Registry.*  ***Side effects:*** *Every year, serious cell-changes are detected in 3000 Norwegian women. 2500 of these will make a full recovery without treatment, but we do not know which ones. If the cell-changes are not treated, they can develop into cervical cancer. 1 out of 12 women who have a pap smear taken, has to take one or more control samples due to abnormal cells being found. The cell-changes can be removed with a minor surgical procedure, but this treatment may increase the risk for late-term miscarriage and premature birth in future pregnancies. The surgery is usually recommended to women with two or more abnormal samples.*  Further information about the Cervical Cancer Screening Programme, including frequently asked questions, can be found at www.kreftregisteret.no/livmorhals |

| **Letter 3 version A**  Letter with recommendation to pursue treatment | **Letter 3 version B**  Letter with recommendation to pursue treatment |
| --- | --- |
| Now imagine that you have received the following letter in the mail from your doctor after having taken the control sample:  **Information concerning discovered cell-changes in the cervix.**  We have now gotten the results from the control samples we took, and these confirm the irregular cells. Such cell-changes can develop into cervical cancer if they are not removed. Therefore we recommend performing a surgical procedure to remove these irregular cells, before they can develop into cervical cancer. Further information, including frequently asked questions, can be found at [www.kreftregisteret.no/livmorhals](http://www.kreftregisteret.no/livmorhals) | Now imagine that you have received the following letter in the mail from your doctor after having taken the control sample:  **Information concerning discovered cell-changes in the cervix.**  We have now gotten the results from the control samples we took, and these confirm the irregular cells. Such cell-changes can develop into cervical cancer if they are not removed. Therefore we recommend performing a surgical procedure to remove these irregular cells, before they can develop into cervical cancer.  ***Benefits and disadvantages to participating in the cervical cancer screening:***  ***Why is it important for me to have a Pap test?*** *Most cell changes in the cervix are caused by Human Papillomaviruses (HPV). HPV infection is very common. HPV occurs both in men and women, and is spread through sexual contact. Most of the HPV infections are harmless and disappear by themselves, but sometimes the infection persists and leads to serious cell changes that can develop into cervical cancer. Treatment is available that effectively reduces the risk of developing cervical cancer. Regular Pap testing (screening) can thereby prevent a large proportion of cancers.*  ***What are the disadvantages of having a Pap test?*** *To participate in a screening program can cause excitement and anxiety until the result of the test is ready. Ask your physician about the advantages and disadvantages of screening or see the web site of The Cancer Registry.*  ***Side effects:*** *Every year, serious cell-changes are detected in 3000 Norwegian women. 2500 of these will make a full recovery without treatment, but we do not know which ones. If the cell-changes are not treated, they can develop into cervical cancer. 1 out of 12 women who have a pap smear taken, has to take one or more control samples due to abnormal cells being found. The cell-changes can be removed with a minor surgical procedure, but this treatment may increase the risk for late-term miscarriage and premature birth in future pregnancies. The surgery is usually recommended to women with two or more abnormal samples.* |

**The U.S. Letters:**

The following 6 information letters were used in the American survey.

| **Letter 1, version A**  Recommendation to have a PAP test taken once every three years. | **Letter 1, version B**  Recommendation to have a PAP test taken once every three years. |
| --- | --- |
| **A Pap test can save your life.**    National guidelines on preventive care from the Centers for Disease Control and Prevention, the American Cancer Society, and other leading health care agencies recommend that women between the ages of 21 and 65 have a Pap test done once every three years. The Pap test can detect changes in your cervical cells that suggest cancer may develop in the future. Effective treatment for these serious cellular changes is available. In line with these national guidelines, public health agencies such as the CDC encourage all women between 21 and 65 who have not had a Pap test taken in the past three years to take this letter to a local health care provider and discuss having a Pap test done.  We recommend that you make an appointment with your general physician / primary care provider to have a Pap test done. If your income is low, or you do not have health insurance, you may be able to get a free or low-cost Pap test through the National Breast and Cervical Cancer Early Detection Program. Please see <http://www.cdc.gov/cancer/cervical/basic_info/screening.htm> for more information. | **A Pap test can save your life.**  National guidelines on preventive care from the Centers for Disease Control and Prevention, the American Cancer Society, and other leading health care agencies recommend that women between the ages of 21 and 65 have a Pap test done once every three years. The Pap test can detect changes in your cervical cells that suggest cancer may develop in the future. Effective treatment for these serious cellular changes is available. In line with these national guidelines, public health agencies such as the CDC encourage all women between 21 and 65 who have not had a Pap test taken in the past three years to take this letter to a local health care provider and discuss having a Pap test done.  We recommend that you make an appointment with your general physician / primary care provider to have a Pap test done. If your income is low, or you do not have health insurance, you may be able to get free or low cost treatment through the National Breast and Cervical Cancer Early Detection Program. Please see <http://www.cdc.gov/cancer/cervical/basic_info/screening.htm> for more information.  **Benefits and disadvantages to participating in cervical cancer screening:**  **Why is it important for me to have a Pap test?** Most cellular changes in the cervix are caused by Human Papillomaviruses (HPV). HPV infection is very common. HPV occurs both in men and women, and is spread through sexual contact. Most of the HPV infections are harmless and disappear by themselves, but sometimes the infections persist and lead to serious changes in cells which can develop into cervical cancer. Effective treatment for these serious cellular changes is available. This treatment reduces the risk of developing cervical cancer. Regular Pap testing (screening) serves to reveal the presence of these serious cellular changes.  **What are the disadvantages of having a Pap test?** Participation in a screening program can cause excitement and/or anxiety until the test results are ready. This is a normal reaction. Ask your physician about the advantages and disadvantages of screening or see the web site of the CDC.  **Side effects:** Every year, serious cellular changes are detected in 300,000 American women. 240,000 of these women will make a full recovery without treatment, but we do not know which ones. If the cellular changes are not treated, they can develop into cervical cancer.1 out of 20 women who have a Pap test done have to get one or more additional Pap tests due to abnormal cells being found. These changed cells can be removed with a minor surgical procedure, but this treatment may increase the risk for late-term miscarriage and premature birth in future pregnancies. The surgery is usually recommended for women who have two or more Pap tests indicating the presence of cellular abnormalities. |

| **Letter 2, version A**  Letter with recommendation for control of cervical pap-smear | **Letter 2, version B**  Letter with recommendation for control of cervical pap-smear |
| --- | --- |
| **Please have a new Pap test done**  Approximately 6 months ago, you had a Pap test done. The result of this Pap test was sent to your doctor/local health care provider, along with the recommendation that you have a new test done in six months. Records indicate that your previous Pap test results revealed the presence of abnormal cells. We are contacting you now to remind you that you should have a second Pap test done as soon as possible.  National health care guidelines recommend that women have a second Pap test done when the first test reveals the presence of irregular cells, or an infection of the human papilloma virus (HPV) in the cervix. While such cellular changes and most HPV-infections usually disappear on their own within 6-12 months, we still recommend having an additional Pap test done.  More information, including answers to frequently asked questions, can be found at the web pages of the CDC. If your income is low, or youdo not have health insurance, you may be able to get a free or low-cost Pap test through the National Breast and Cervical Cancer Early Detection Program. Please see <http://www.cdc.gov/cancer/cervical/basic_info/screening.htm> for more information. | **Please have a new Pap test done**  Approximately 6 months ago, you had a Pap test done. The result of this Pap test was sent to your doctor/local health care provider, along with the recommendation that you have a new test done in six months. Records indicate that your previous Pap test results revealed the presence of abnormal cells. We are contacting you now to remind you that you should have a second Pap test done as soon as possible.  National health care guidelines recommend that women have a second Pap test done when the first test reveals the presence of irregular cells, or an infection of the human papilloma virus (HPV) in the cervix. While such cellular changes and most HPV-infections usually disappear on their own within 6-12 months, we still recommend having an additional Pap test done.  More information, including answers to frequently asked questions, can be found at the web pages of the CDC. If your income is low, or you do not have health insurance, you may be able to get a free or low-cost Pap test through the National Breast and Cervical Cancer Early Detection Program. Please see <http://www.cdc.gov/cancer/cervical/basic_info/screening.htm> for more information.  **Benefits and disadvantages to participating in cervical cancer screening:**  **Why is it important for me to have a Pap test?** Most cellular changes in the cervix are caused by Human Papillomaviruses (HPV). HPV infection is very common. HPV occurs both in men and women, and is spread through sexual contact. Most of the HPV infections are harmless and disappear by themselves, but sometimes the infections persist and lead to serious changes in cells which can develop into cervical cancer. Effective treatment for these serious cellular changes is available. This treatment reduces the risk of developing cervical cancer. Regular Pap testing (screening) serves to reveal the presence of these serious cellular changes.  **What are the disadvantages of having a Pap test?** Participation in a screening program can cause excitement and/or anxiety until the test results are ready. This is a normal reaction. Ask your physician about the advantages and disadvantages of screening or see the web site of the CDC.  **Side effects:** Every year, serious cellular changes are detected in 300,000 American women. 240,000 of these women will make a full recovery without treatment, but we do not know which ones. If the cellular changes are not treated, they can develop into cervical cancer.1 out of 20 women who have a Pap test done have to get one or more additional Pap tests due to abnormal cells being found. These changed cells can be removed with a minor surgical procedure, but this treatment may increase the risk for late-term miscarriage and premature birth in future pregnancies. The surgery is usually recommended for women who have two or more Pap tests indicating the presence of cellular abnormalities. |

| **Letter 3 version A**  Letter with recommendation to pursue treatment | **Letter 3 version B**  Letter with recommendation to pursue treatment |
| --- | --- |
| Now imagine that you have received the following letter in the mail from your primary health care provider after having had a second Pap test done:  **Information concerning the discovery of cellular changes in your cervix.**  Recently, you had a Pap test done. We have now gotten the results from your second Pap test, and these results confirm the presence of irregular cells. Such changes within the cells can develop into cervical cancer if they are not removed. We recommend that you contact us in order to make arrangements for the minor surgical procedure needed to remove these irregular cells before they can develop into cervical cancer  More information, including answers to frequently asked questions, can be found at the web pages of the CDC. If your income is low, or you do not have health insurance, you may be able to get free or low cost treatment through the National Breast and Cervical Cancer Early Detection Program. Please see <http://www.cdc.gov/cancer/cervical/basic_info/screening.htm> for more information. | Now imagine that you have received the following letter in the mail from your primary health care provider after having had a second Pap test done:  **Information concerning the discovery of cellular changes in your cervix.**  Recently, you had a Pap test done. We have now gotten the results from your second Pap test, and these results confirm the presence of abnormal cells. Such changes within the cells can develop into cervical cancer if they are not removed. In accordance with national guidelines, we recommend that you contact us in order to make arrangements for the minor surgical procedure needed to remove these irregular cells before they can develop into cervical cancer  If your income is low, or youdo not have health insurance, you may be able to get free or low cost treatment through the National Breast and Cervical Cancer Early Detection Program. Please see <http://www.cdc.gov/cancer/cervical/basic_info/screening.htm> for more information.  **Benefits and disadvantages to participating in cervical cancer screening:**  **Why is it important for me to have a Pap test?** Most cellular changes in the cervix are caused by Human Papillomaviruses (HPV). HPV infection is very common. HPV occurs both in men and women, and is spread through sexual contact. Most of the HPV infections are harmless and disappear by themselves, but sometimes the infections persist and lead to serious changes in cells which can develop into cervical cancer. Effective treatment for these serious cellular changes is available. This treatment reduces the risk of developing cervical cancer. Regular Pap testing (screening) serves to reveal the presence of these serious cellular changes.  **What are the disadvantages of having a Pap test?** Participation in a screening program can cause excitement and/or anxiety until the test results are ready. This is a normal reaction. Ask your physician about the advantages and disadvantages of screening or see the web site of the CDC.  **Side effects:** Every year, serious cellular changes are detected in 300,000 American women. 240,000 of these women will make a full recovery without treatment, but we do not know which ones. If the cellular changes are not treated, they can develop into cervical cancer.1 out of 20 women who have a Pap test done have to get one or more additional Pap tests due to abnormal cells being found. These changed cells can be removed with a minor surgical procedure, but this treatment may increase the risk for late-term miscarriage and premature birth in future pregnancies. The surgery is usually recommended for women who have two or more Pap tests indicating the presence of cellular abnormalities. |
